# Engineered acetylcholinesterase-loaded dissolvable microneedles mitigate dermal toxicity by targeting trichlorfon binding and metabolic pathway modulation

**DOI:** 10.1016/j.mtbio.2026.103243

**Published:** 2026-05-14

**Authors:** Shuoqi Jiang, Zi-Wei Zheng, Qiuya Gu, Jian-Xin Li, Xiaobin Yu, Zhuangwei Zhang

**Affiliations:** a Digital Industry Research Institute, Zhejiang Wanli University, No.8 South Qian Hu Road, Ningbo, Zhejiang, China; b Key Laboratory of Industrial Biotechnology, Ministry of Education, School of Biotechnology, Jiangnan University, 1800 Li-Hu Road, Bin-Hu District, Wuxi, Jiangsu, China; c Central Laboratory of the Medical Research Center, The First Affiliated Hospital of Ningbo University, Ningbo, Zhejiang, China; d State Key Laboratory of Analytical Chemistry for Life Science, Collaborative Innovation Centre of Chemistry for Life Sciences, Jiangsu Key Laboratory of Advanced Organic Materials, School of Chemistry and Chemical Engineering, Nanjing University, Nanjing, Jiangsu, China

**Keywords:** Acetylcholinesterase, Trichlorfon, Microneedles, Targeted detoxification, Exogenous enzyme therapy

## Abstract

Trichlorfon (TCF), a widely used organophosphorus pesticide for crop protection, poses severe dermal toxicity risks as it readily penetrates the epidermal barrier and metabolizes into the more toxic dichlorvos (DDVP), triggering neurotoxicity, oxidative stress and inflammatory damage in exposed individuals. As a promising stoichiometric bioscavenger for pesticide detoxification, natural acetylcholinesterase (AChE) is still plagued by bottlenecks in thermal stability and transdermal delivery efficiency. To address these issues, this study employed a previously engineered high-activity and thermostable AChE variant (*Cp*A-M5), evaluated its detoxification efficacy and mechanisms both *in vitro* and *in vivo*, and further developed a *Cp*A-M5-loaded dissolvable microneedle system (*Cp*A-M5-MN) for targeted dermal detoxification. *In vitro* assays demonstrated that *Cp*A-M5 effectively restored endogenous AChE activity, scavenged ROS, reduced inflammation and alleviated apoptosis in TCF-exposed cells. Enzyme kinetics and molecular interaction analyses confirmed irreversible binding between *Cp*A-M5 and TCF/DDVP, with DDVP showing higher binding affinity (*K*_*D*_ = 9.23 × 10^−6^ M, *Ki* = 15.56 μM) and complex stability (*K*_*a*_ = 2450.191 M^−1^, *ΔG* = −39.37 ± 1.48 kcal/mol). *In vivo*, the *Cp*A-M5-loaded microneedle system achieved targeted transdermal delivery, effectively overcoming the limitations of low permeability and rapid degradation of free enzymes. Pathological and metabolomic analyses demonstrated that *Cp*A-M5-MN reduced skin residual TCF/DDVP levels, restored local oxidative-inflammatory homeostasis, and regulated glutathione and amino acid metabolic pathways, thereby achieving synergistic detoxification and metabolic repair effects. Collectively, this work presents a promising targeted detoxification platform for pesticide-induced dermal injury, and provides new insights into the clinical translation of engineered enzyme-based bioscavengers.

## Highlights

• An engineered AChE variant (*Cp*A-M5) acts as an efficient enzyme-based bioscavenger.
• *Cp*A-M5 exerts potent therapeutic efficacy in mitigating TCF-induced toxicity.
• Molecular interaction assays verify the binding mechanism between *Cp*A-M5 and TCF/DDVP.
• *Cp*A-M5-loaded dissolvable microneedle system enables targeted dermal detoxification.
• *Cp*A-M5-MN achieves synergistic detoxification and metabolic repair.

## Graphical abstract

**Figure:**
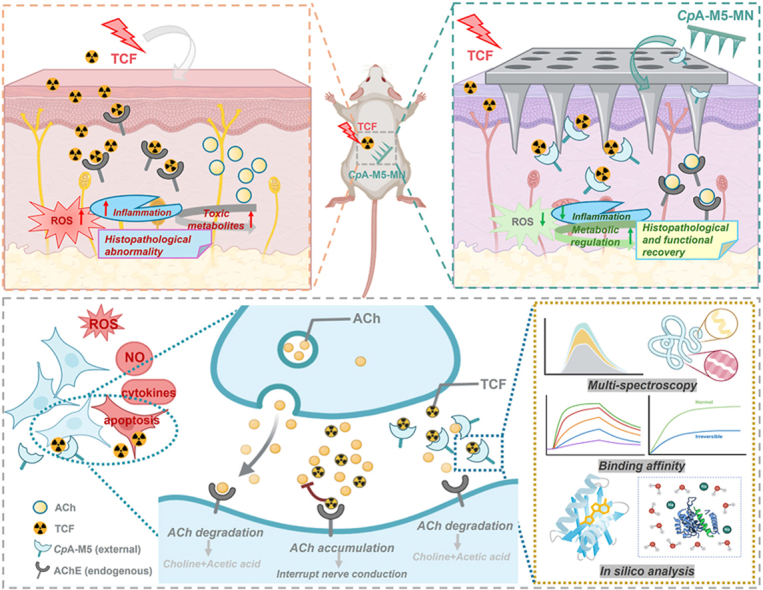

## 1 Introduction

Pesticides, as widely used agricultural and public health agents, pose significant threats to human health through multiple exposure routes, among which dermal absorption is recognized as a major pathway—especially for low-volatile organophosphorus pesticides (OPPs) such as trichlorfon (TCF) and malathion [1]. Farmers, pesticide applicators, and individuals in occupational or environmental settings are particularly vulnerable to dermal exposure, such as during pesticide spraying, mixing and dilution, or when personal protective equipment is insufficient, and lipophilic pesticide molecules can readily penetrate the epidermal barrier and enter the systemic circulation [2,3]. Notably, short-term, low-dose, and localized dermal contact usually leads to mild acute poisoning characterized by local symptoms, including dry skin, pruritus, abnormal sweating, and muscle fatigue [4,5]. In contrast, prolonged or high-level exposure causes a gradual reduction in serum cholinesterase activity and gives rise to chronic health consequences due to cumulative skin penetration [6]. Upon dermal absorption, OPPs not only inhibit acetylcholinesterase (AChE) activity to induce neurotoxicity but also trigger excessive production of reactive oxygen species (ROS), disrupting the oxidant-antioxidant balance and causing oxidative damage to proteins, lipids, and nucleic acids [7,8]. This oxidative stress further amplifies inflammatory responses by promoting the release of pro-inflammatory cytokines, ultimately leading to skin barrier dysfunction, cellular apoptosis, and even systemic pathological damage [9,10]. Given the global burden of pesticide-induced toxicity, with an estimated 385 million annual poisonings and 11,000 deaths worldwide [11], developing effective strategies to mitigate dermal pesticide exposure damage is an urgent public health need.

Chemical stoichiometric bioscavengers, including AChE, butyrylcholinesterase (BChE), and carboxylesterases (CarE), are promising candidates for targeted pesticide detoxification [12]. Among these, AChE exhibits unique advantages as it is more stereoselective than BChE, binding preferentially to the highly toxic stereoisomers of OPPs [13], and provides superior protection against OPPs exposure at equivalent doses in animal models [14]. However, AChE faces distinct challenges that hinder its direct application as a bioscavenger, as its critical role in regulating cholinergic neural transmission enables freely circulating AChE to disrupt neurotransmission and induce severe side effects [15]. To address this limitation, researchers have immobilized AChE onto red blood cell (RBC) membranes to fabricate nanoparticles (RBC-NPs), which retain enzymatic activity while circumventing systemic neurotoxicity [16]. Additionally, AChE shares common limitations with other stoichiometric bioscavengers: natural sources are scarce, and recombinant expression, even when combined with protein engineering strategies [17], remains inefficient, thereby contributing to high production costs. Its “consumable” nature, characterized by irreversible binding to OPPs in a molar ratio, also necessitates large doses for effective detoxification. Furthermore, exogenous AChE suffers from short *in vivo* half-lives due to proteolytic degradation, inefficient delivery to dermal exposure sites, and potential immunogenicity [18]. Despite advancements in AChE optimization *via* molecular engineering [19,20], these limitations persist, underscoring the need for integrated strategies to simultaneously address delivery, safety, and efficacy.

To address these bottlenecks, three complementary and targeted strategies have garnered considerable attention. First, microneedle (MN) technology has emerged as a revolutionary transdermal delivery platform for macromolecular agents. The micron-scale needle array can painlessly penetrate the stratum corneum to create reversible microchannels, enabling direct delivery of therapeutic enzymes to the dermal layer [21]. This approach not only bypasses the epidermal barrier but also avoids first-pass metabolism and gastrointestinal degradation, and maintains stable local drug concentrations, effectively resolving the inefficiency of localized delivery of exogenous AChE [22,23]. Second, enzyme-based bioscavengers, operating *via* their inherent reaction mechanism, specifically bind to the active groups of pesticides such as through covalent interaction with the active serine residues of OPPs or catalyze their degradation. Functioning as “biological scavengers”, they neutralize toxicants directly at the dermal exposure site before these substances penetrate into the systemic circulation, thereby circumventing the non-targeted toxicity of traditional detoxifiers to host tissues [24,25]. Third, prior molecular engineering of AChE has yielded improvements. Through structure-guided rational design and semi-rational design strategies, the engineered AChE variant *Cp*A-M5 exhibits enhanced thermal stability, improved catalytic efficiency, and targeted depletion of TCF, effectively overcoming the limitations of natural AChE in terms of stability and efficacy [26,27]. Utilizing MN-mediated transdermal delivery to target engineered AChE variants to dermal exposure sites provides a synergistic solution to the core challenges of enzyme-based pesticide detoxification.

In this study, we aim to develop a microneedle system loaded with the engineered AChE variant *Cp*A-M5 for targeted detoxification of TCF-induced dermal toxicity. We first evaluated *Cp*A-M5's protective effects on TCF-exposed RAW 264.7 and HT-22 cells. Subsequently, multispectral analysis, biomolecular interaction assays, and *in silico* approaches were used to investigate the molecular interaction mechanism between *Cp*A-M5 and TCF as well as its toxic metabolite dichlorvos (DDVP), verifying catalytic specificity and binding affinity. The *in vivo* efficacy of the microneedle system and its core regulatory pathways were validated in TCF-exposed mice by assessing skin pathological damage, biochemical marker levels, and untargeted metabolomics. Collectively, this study seeks to provide a novel and efficient exogenous enzyme delivery strategy for pesticide-induced dermal toxicity, clarify the underlying detoxification mechanisms, and lay a foundation for the clinical translation of engineered enzyme-based bioscavengers in dermal protection and emergency detoxification scenarios.

## 2 Materials and methods

### 2.1 Chemicals

*Escherichia coli* SHuffle T7 was used as the host strain for protein expression and *E. coli* JM109 was employed as the host for gene cloning. Plasmid pColdⅡ was obtained from the laboratory collection. Compounds S-acetylthiocholine iodide (ATCh) and 5,5′-dithio bis-(2-nitrobenzoic acid) (DTNB) were purchased from InnoChem (Beijing, China) and Titan Scientific (Shanghai, China), respectively. Obidoxime dichloride (cat. No. HY-W011108) was obtained from MedChemExpress (MCE). Human recombinant AChE (HuAChE) was purchased from Sigma-Aldrich (cat. No. C1682). Pierce High Capacity Endotoxin Removal Resin was purchased from Thermo Scientific. Chromogenic tachypleus amebocyte lysate (TAL) test kit (cat. No. EC64405) was purchased from Xiamen Bioendo Technology Co., Ltd. (Xiamen, China). Dulbecco's Modified Eagle Medium (DMEM) (cat. No. BC-M-005) and fetal bovine serum (FBS) (cat. No. BC-SE-FBS08) were obtained from SenBeiJia Biological Technology Co., Ltd. (Jiangsu, China). Enhanced Cell Counting Kit-8 (CCK-8) (cat. No. BL1055C), Reactive Oxygen Species (ROS) assay kit (cat. No. BL714A), Nitric Oxide (NO) assay kit (cat. No. BL1204A) and Annexin V-FITC Apoptosis Detection Kit (cat. No. BL107A) were purchased from Biosharp Biotechnology (Anhui, China). 96-well plates (cat. No. 713011) were purchased from NEST Biotechnology Co., Ltd. (Wuxi, China). Trichlorfon (BWN0122-2016, GBW(E)083,205) and Dichlorvos (BWN0102-2016, GBW(E)083,103) were provided by North Weiye Measurement Technology Research Institute (Beijing, China). SelectCore HLB solid-phase extraction (SPE) cartridges were purchased from NanoChrom (Suzhou, China). Mouse interleukin-6 (IL-6) ELISA kit (cat. No. SBJ-M0657), mouse interleukin-1β (IL-1β) ELISA kit (cat. No. SBJ-MM0027), mouse tumor necrosis factor-α (TNF-α) ELISA kit (cat. No. SBJ-M0030), mouse interferon-γ (IFN-γ) ELISA kit (cat. No. SBJ-M0038), mouse immunoglobulin A (IgA) ELISA kit (cat. No. SBJ-M0007) and mouse immunoglobulin M (IgM) ELISA kit (cat. No. SBJ-M0006) were purchased from SenBeiJia Biological Technology Co., Ltd. (Nanjing, China). Hematoxylin-eosin (HE) staining kit (cat. No. C0105S) and Masson's trichrome staining kit (cat. No. C0189S) were obtained from Beyotime Bio Inc. (Shanghai, China). Malondialdehyde (MDA) assay kit (cat. No. BL1481B), total antioxidant capacity (T-AOC) kit (cat. No. BL858B), catalase (CAT) kit (cat. No. BL855B), total superoxide dismutase (SOD) activity assay kit (cat. No. BL901A), urea nitrogen (BUN) assay kit (cat. No. BL1484B), creatinine (CRE) assay kit (cat. No. BL890B), aspartate aminotransferase (AST) activity assay kit (cat. No. SBJ-M0657), alanine aminotransferase (ALT) activity assay kit (cat. No. BL1409B), and fluorescein isothiocyanate (FITC) (cat. No. BS096) were purchased from Biosharp Biotechnology (Anhui, China). Hyaluronic acid (HA, molecular weight: 30-45 kDa) (cat. No. H924870), polyvinyl alcohol (PVA, cat. No. P875084), and orthophosphoric acid (85%) (cat. No. P816337) were obtained from Macklin (Shanghai, China). Methanol and acetonitrile were of chromatographic grade, and all other reagents were of analytical grade.

### 2.2 Protein expression and purification

The recombinant protein *Cp*A-M5 used in this study was an engineered variant derived from our previously reported thermostable and catalytically enhanced mutant M5 of *Culex pipiens* AChE [27]. Recombinant *E. coli* was cultured in Luria-Bertani (LB) medium at 37 °C, 200 rpm until OD_600_ = 0.6. Protein expression was induced with isopropyl β-D-1-thiogalactopyranoside (IPTG) and maintained overnight at 16 °C. After sonication (JY92-IIN, Scientz Biotechnology), the soluble fraction was purified using Ni-charged resin FF (GenScript). Imidazole was removed by washing 5 times with 100 mM PBS (pH 7.4) in an ultrafiltration tube. Endotoxin was removed using Pierce high-capacity endotoxin removal resin, and residual endotoxin was confirmed to be < 0.1 EU/ml *via* TAL test kit. The protein solution was filtered through a 0.22 μm syringe filter and frozen for storage.

### 2.3 Cell culture and viability assay

HT-22 and RAW 264.7 cells were adherently cultured in DMEM supplemented with 10% FBS and 1% penicillin-streptomycin, and incubated at 37 °C with 5% CO_2_ in a humidified incubator. After 12 h of stabilization, cells were treated with trichlorfon (TCF, 7.77 – 7.77 × 10^5^ nM) at various concentrations or *Cp*A- M5 enzyme solution (10, 50, 100, 200, 500, 1000 μg/mL) for 8, 16, or 24 h.

Cells were divided into four groups: Control (serum-free DMEM), Model (pesticide at IC_50_ + serum-free DMEM), Prophylactic (100 μg/mL recombinant enzyme M5 pretreatment + pesticide at IC_50_ + serum-free DMEM), and Curative (pesticide at IC_50_ pretreatment + 100 μg/mL recombinant enzyme M5 + serum-free DMEM). Subsequently, 10 μL CCK-8 solution was added to each well, incubated at 37 °C for 1 h, and the optical density was measured at 450 nm using an automatic microplate reader.

### 2.4 Cell AChE activity assay

AChE activity was determined *via* a slightly modified Ellman's method. Briefly, treated grouped cells in 6-well plates were washed with PBS, scraped, and resuspended in equal-volume PBS. Cells were sonicated on ice for lysis, and the centrifuged supernatant was normalized for total protein. A 90 μL aliquot of the supernatant was mixed with 110 μL enzyme activity detection reagent in a 96-well plate, and kinetic assays were performed at 412 nm. All samples were run in triplicate and expressed as a percentage of the untreated control.

### 2.5 Intracellular NO detection and immune factor detection

NO production was indirectly measured by detecting nitrite in the supernatant of RAW 264.7 cells. RAW 264.7 cells in 96-well plates were grouped and treated, then 50 μL of supernatant was mixed with equal volumes of Griess Reagent I and II. After incubation at room temperature, absorbance was measured at 540 nm.

Levels of IL-6, IL-1β, TNF-α, and IFN-γ in RAW 264.7 cell supernatant were detected by ELISA. After grouped treatment of cells in 96-well plates, supernatant was collected, and absorbance was measured at 450 nm according to the kit instructions.

### 2.6 Intracellular ROS detection

ROS levels were detected using a ROS detection kit. Grouped cells in 6-well plates were treated, then incubated with diluted H_2_DCFDA working solution at 37 °C in the dark for 30 min. After PBS washing, green fluorescence was observed and captured under a fluorescence microscope, and ROS levels were quantified by flow cytometry (CytoFLEX, Beckman Coulter, Germany).

### 2.7 Cell apoptosis detection

Cell apoptosis was detected using an Annexin V-FITC/PI Apoptosis Detection Kit. Treated grouped cells in 6-well plates were washed twice with PBS, then incubated with 500 μL binding buffer containing equal amounts of Annexin V-FITC and PI at room temperature in the dark for 5 min. Cells were observed under a fluorescence microscope. For flow cytometry analysis, stained cells were collected, resuspended in dye-containing binding buffer, and analyzed within 1 h.

### 2.8 High-performance liquid chromatography (HPLC) analysis

SelectCore HLB SPE cartridges were activated with 1–2 column volumes of methanol and equilibrated with 1–2 column volumes of water. TCF-supplemented DMEM medium was incubated at 37 °C for 12 and 24 h, then loaded onto the SPE cartridges. The flow-through was discarded, and the cartridges were rinsed with 1–2 column volumes of water (rinse solution discarded). Elution was performed with 5 mL of acetonitrile, and the eluate was filtered through a 0.22 μm membrane prior to instrumental analysis. TCF and DDVP standards were accurately weighed and prepared as 1.0 mg/mL stock solutions in acetonitrile, then filtered through a 0.22 μm membrane for analysis. HPLC analysis was performed using a system equipped with an autosampler, micropump, on-line vacuum degasser, and temperature-controlled column compartment (Shimadzu-LC-20 A T, Japan). Chromatographic separation was achieved on a C18 column (150 mm × 4.6 mm, 5 μm particle size) at 30 °C. The mobile phase consisted of acetonitrile:water (15:85, *v*/*v*), with water adjusted to pH 3 using phosphoric acid; it was filtered and degassed prior to use. The flow rate was set at 1 mL/min, injection volume at 10 μL, and detection wavelength at 200 nm.

### *2.9* *In vitro* interaction assay

#### *2.9.1* *In vitro* enzyme inhibition assay

In brief, 90.0 μL of appropriately diluted *Cp*A-M5 enzyme solution (0.01–0.06 mg/mL) was mixed in a 200 μL reaction mixture containing 1.0 mM ATCh (50 μL) and 0.1 mM DTNB (50 μL). For inhibitor treatment, 10 μL of gradient TCF or DDVP solutions (0 - 60 μM) were added to the system. For the control group, 10 μL of methanol was added instead of inhibitor solution, giving a final methanol concentration of 5% (*v*/*v*). After incubation at room temperature for 30 min, residual enzyme activity was determined as described previously [26]. All assays were performed in three independent experiments.

#### 2.9.2 Inhibition kinetics of *Cp*A-M5

##### 2.9.2.1 Second-order inhibition rate constant (*k*_*i*_)

The bimolecular inhibition rate constants (*k*_*i*_) of TCF and DDVP against *Cp*A-M5 were determined as previously described [28], by monitoring the time-dependent inhibition of enzyme activity at 37 °C in the presence of substrate. Briefly, residual enzyme activity was continuously recorded after the addition of inhibitors, which were used at sufficiently high concentrations to establish pseudo-first-order reaction conditions. The *k*_*i*_ value was calculated using pseudo-first-order kinetic analysis according to the equation:

(1) $$k_{i}=\frac{1}{\left[OP\right]t}\ln\frac{v_{0}}{v}$$

where [OP] is the initial concentration of the tested organophosphate, and *v*_*0*_ and *v* represent the reaction velocity at time zero and time t, respectively.

##### 2.9.2.2 Spontaneous reactivation rate constant (*k*_*r*_)

Spontaneous reactivation kinetics were measured using a modified protocol based on a previously reported method [29]. *Cp*A-M5 was pre-inhibited with TCF or DDVP, and residual free inhibitor was removed by ultrafiltration (10 kDa molecular weight cutoff). The enzyme was then incubated at 37 °C, and the recovery of enzymatic activity was monitored at designated time intervals. The spontaneous reactivation rate constant (*k*_*r*_) was obtained by fitting the reactivation curve to a single exponential function:

(2) $$\ln\left(1-\frac{EI}{E}\right)=-k_{r}t$$

where EI is the activity of the inhibited enzyme solution, E is the activity of the control enzyme solution, and t is the time (in hours) since inhibition.

##### 2.9.2.3 Aging rate constant (*k*_*a*_)

Aging kinetics were determined as reported previously with minor modifications [30]. After inhibition with TCF or DDVP, unbound inhibitor was eliminated by ultrafiltration. At successive time points, the inhibited *Cp*A-M5 was incubated with 50 μM obidoxime at 37 °C for 30 min, and the remaining reactivatable enzymatic activity was measured. The aging rate constant (*k*_*a*_) was calculated from the exponential decay of reactivatable activity:

(3) $$\ln\left(\frac{EIR}{ER}\right)=-k_{a}t$$

where ER is the reactivated control enzyme activity, EIR is the inhibited enzyme activity after reactivation, and t is the time (in hours) post-inhibition.

#### 2.9.3 Fluorescence spectroscopic assay

Fluorescence titration was conducted to characterize the interaction changes between *Cp*A-M5 and TCF/DDVP. The *Cp*A-M5 solution (0.1 mg/mL) was continuously titrated with TCF or DDVP (2.5 mM, 5 μL), and fluorescence spectra were collected after sufficient incubation at room temperature. The excitation wavelength was set at 280 nm, and emission wavelengths ranged from 300 to 500 nm with both excitation and emission slit widths of 5 nm. Synchronous fluorescence spectra were recorded at constant wavelength intervals (Δλ) of 15 nm and 60 nm, respectively. PBS solution was used as the blank background for baseline correction.

The fluorescence quenching mechanism was analyzed using the Stern-Volmer equation. The binding constant (*K*_*a*_) and the number of binding sites (n) were determined *via* double logarithmic regression equation. The change in Gibbs free energy (Δ*G*°) was calculated using the following formulas:

(4) $$\frac{F_{0}}{F}=1+K_{sv}\left[Q\right]=1+K_{q}\tau_{0}\left[Q\right]$$

(5) $$\mathrm{lg}\frac{F_{0}-F}{F}=\mathrm{lg}\;K_{a}+n\;\mathrm{lg}\left[Q\right]$$

(6) $$\Delta G{}^{\circ}=-\text{RTln}K_{a}$$

where *F*_*0*_ and *F* represent the fluorescence intensities of *Cp*A-M5 in the absence and presence of quencher, respectively; *K*_*sv*_ is the Stern-Volmer constant; *K*_*q*_ is the quenching constant; *τ*_*0*_ is the average fluorescence lifetime of typical biomacromolecules (10^−8^ s); [*Q*] is the quencher concentration; R is the gas constant (8.314 J/mol·K); and T is the absolute temperature (298 K).

#### 2.9.4 Circular dichroism (CD) spectrum measurement

CD spectroscopy (JASCO-J-1700, Japan) was employed to investigate the changes in the secondary structure of *Cp*A-M5. The purified enzyme solution was diluted to 0.15 mg/mL in 20 μM PBS buffer (pH 8.0) and incubated with TCF or DDVP at 25 °C for 30 min to explore structural changes at different conditions. The CD spectra in the ultraviolet region (190–260 nm) were recorded as the average of three scans at a scan speed of 100 nm/min and a resolution of 1 nm. The blank spectrum of the buffer solution was subtracted for correction.

#### 2.9.5 Biolayer interferometry (BLI) affinity assay

BLI was used to characterize the binding affinity between TCF/DDVP and *Cp*A-M5 by measuring the optical interference shift caused by mass changes of the protein-coated sensor chip during analyte binding. *Cp*A-M5 protein was biotinylated using EZ-Link™ Sulfo-NHS-LC-LC-Biotin, and unreacted biotin was removed using a 3 kDa ultrafiltration tube with a volume of 0.5 mL. Streptavidin (SA) biosensors were prewetted in 10 mM PBS (0.02% Tween-20) for 30 min and the same buffer was used as the baseline buffer. *Cp*A-M5 (100 μg/mL) and TCF/DDVP at different concentrations (50 μM, 100 μM, 500 μM, 1000 μM) were loaded into sample plates, with data acquisition parameters set at 25 °C and 1000 rpm.

The measurement procedure included the following steps: sensor immersion in baseline (180 s), protein loading (300 s), re-immersion in baseline (180 s), association (360 s), and dissociation (240 s). The equilibrium dissociation constant (*K*_*D*_) was fitted and calculated using Octet Analysis Studio 12.2 software (ForteBio, CA, USA) based on a 1:1 binding model.

### 2.10 Molecular dynamics (MD) simulation

MD simulations were performed to evaluate the structural stability and dynamic behavior of the *Cp*A-M5/DDVP complex using the Desmond package (v7.2). The initial structure of the complex was solvated in an orthorhombic box with the SPC water model under the OPLS_2005 force field. The system was neutralized by adding Na^+^ and Cl^−^ ions and was subsequently energy-minimized and equilibrated under NVT and NPT ensembles. Production simulations were conducted under the NPT ensemble at 300 K for 100 ns, with trajectories recorded every 100 ps The root mean square deviation (RMSD) and root mean square fluctuation (RMSF) were analyzed to assess the conformational stability and residual flexibility of the complex.

### 2.11 Preparation of *Cp*A-M5-MNs

FITC labeling of *Cp*A-M5 was confirmed by thin-layer chromatography (TLC). Briefly, *Cp*A-M5 was dissolved in 10 mM PBS and reacted with FITC at a molar ratio of 1:5 for 6 h at 4 °C in the dark. Unbound FITC and buffer were removed by ultrafiltration. The reaction mixture, along with unlabeled *Cp*A-M5 and FITC solutions, was separately spotted onto a silica gel plate. After drying under cold air, the plate was developed in ethanol–water (3:1, v/v). Upon solvent migration to the top, the plate was air-dried, and fluorescence was visualized and recorded under 365 nm UV light using a gel imaging system.

**Fig. 1:**
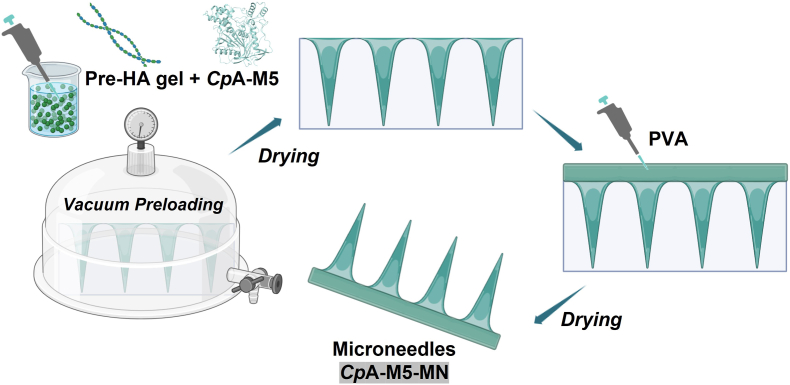
Schematic diagram of the preparation process of *Cp*A-M5 dissolving microneedles.

*Cp*A-M5-loaded microneedles (*Cp*A-M5-MNs) were fabricated using a two-step filling procedure as follows (Fig. 1):

**Tip Layer Preparation**: *Cp*A-M5 (5 mg/mL) and hyaluronic acid (HA, 15% w/v) were dissolved in PBS (pH 7.4) and vortex-mixed for 10 min to obtain a homogeneous solution. The mixture was applied to a custom polydimethylsiloxane (PDMS) mold (15 × 15 array of quadrangular pyramids; needle length: 800 μm, base: 360 × 360 μm, tip spacing: 720 μm), degassed under vacuum (−0.08 MPa) for 15 min, and centrifuged (4 °C, 4000 rpm, 5 min) to fill the needle cavities. Excess solution was removed, and the mold was dried at 4 °C for 12 h to solidify the tip layer.

**Base Layer Preparation**: A 10% (w/v) polyvinyl alcohol (PVA) aqueous solution was coated onto the solidified tip layer, followed by centrifugation (4000 rpm, 5 min) to ensure layer adhesion. The patch was dried at 4 °C for 12 h to form the base layer.

**Demolding and Storage**: The dried microneedle patch was carefully peeled from the PDMS mold and stored in a desiccator at 4 °C.

### 2.12 Characterization of *Cp*A-M5-MNs

#### 2.12.1 Morphological characterization

**Bright-field and fluorescence microscopy**: *Cp*A-M5-MNs were horizontally placed on a microscope stage. Macroscopic morphology was examined under bright-field mode using an optical microscope. To visualize the distribution of FITC-labeled *Cp*A-M5, fluorescence images were acquired under 488 nm excitation, and the quadrangular pyramid morphology, tip integrity, and localization of *Cp*A-M5 in the tip layer were analyzed.

**Scanning electron microscopy (SEM)** (**S-4800, Hitachi, Japan)**: Microneedle patches were vertically mounted on conductive tape and sputter-coated with an 8 nm gold layer (Hitachi E−1045). The microstructure of microneedle tips was observed at an accelerating voltage of 15 kV and magnifications of 20–150× to assess tip sharpness and the integrity of the tip–base interface.

**Fourier transform infrared (FT-IR) spectroscopy** (**Nicolet 6700, Thermo Fisher Scientific, USA)**: HA, *Cp*A-M5, and the HA + *Cp*A-M5 tip layer were separately ground, dried, mixed with KBr powder (1:100, w/w), and pressed into pellets. FT-IR spectra were recorded from 400 to 4000 cm^−1^. Characteristic bands of the microneedle components were identified by comparing spectral shifts among the samples.

#### 2.12.2 Mechanical properties

The mechanical strength of the full *Cp*A-M5-MN array was evaluated using a texture analyzer. The patch was vertically fixed with tips facing upward. A cylindrical flat probe was used in compression mode with a loading speed of 0.1 mm/s, a trigger force of 0.05 N, and a preset displacement of 800 μm. The probe descended vertically until the set displacement was reached or fracture occurred, and stress–displacement curves were recorded in real time. All tests were performed in triplicate.

#### 2.12.3 Stability evaluation

The storage stability of *Cp*A-M5-MNs was evaluated by monitoring the enzymatic activity of both the free enzyme and the microneedle-incorporated form. The free enzyme (*Cp*A-M5, 1 mg/mL in PBS, pH 7.4) was stored in light-protected tubes at 4 °C. The microneedle patches were sealed in aluminum foil bags and stored at 4 °C and 25 °C, with three parallel samples for each group and condition. At predetermined time points (0, 7, 14, 21, and 28 days), samples were taken for activity analysis: the free enzyme solution was directly assayed, while a microneedle patch was dissolved in 1 mL of PBS for measurement.

#### *2.12.4* *In vitro* dissolution performance

The *in vitro* dissolution behavior of *Cp*A-M5-MNs was dynamically monitored using fluorescence microscopy with an agarose gel model. Agarose powder (0.8 g) was dispersed in 50 mL PBS, dissolved by microwave heating, and cast into a 5 mm-thick mold to form a 2.0% (w/v) skin-simulating gel. Microneedle patches were inserted vertically into the gel, and fluorescence images (488 nm excitation) were captured at 0, 1, and 3 min to track dissolution.

### 2.13 Insertion test of *Cp*A-M5-MNs

#### 2.13.1 Insertion performance analysis

Skin Sample Preparation: The dorsal skin of male ICR mice (6 weeks old) was shaved and disinfected. FITC-labeled *Cp*A-M5-MNs were inserted vertically for 5 min. After removal, the treated skin tissue was excised, fixed in 4% paraformaldehyde for 24 h, dehydrated through an ethanol gradient, paraffin-embedded, and longitudinally sectioned.

Histological Staining: For H&E staining, sections were deparaffinized in xylene, rehydrated through a descending ethanol series, stained with hematoxylin and eosin, dehydrated, cleared, and mounted. For fluorescence staining, rehydrated sections were incubated with DAPI (1 μg/mL, 10 min) to visualize nuclei, then mounted with anti-fade medium.

Confocal Microscopy (Zeiss-LSM 710, German): FITC-labeled *Cp*A-M5-MNs were inserted into *ex vivo* porcine skin (1.5 mm thick) under 10 N pressure for 3 min. After removal, tissues were fixed and imaged by confocal microscopy with 488 nm excitation. Z-stacks were acquired (20 μm step size, 1.8 mm total depth), and puncture channels were 3D-reconstructed using LAS X software to determine insertion depth.

#### 2.13.2 Skin irritation determination

Skin irritation and recovery were evaluated by macroscopic observation. *Cp*A-M5-MN patches were applied to shaved dorsal skin for 3 min. The puncture sites were photographed 3–10 min after patch removal to document micropore closure.

#### *2.13.3* *In vitro* release study

The *in vitro* release profile of FITC-labeled *Cp*A-M5 was evaluated using Franz diffusion cells. Full-thickness mouse dorsal skin was prepared by removing hair and subcutaneous fat. *Cp*A-M5-MNs were applied to the skin surface (5 N/needle, 5 min) and mounted in Franz cells with the dermis facing the receptor chamber (PBS, pH 7.4, 37 °C, 150 rpm). The control group received free FITC-*Cp*A-M5 solution. At 0, 2, 4, 6, and 8 h, the application area was excised, dissolved in PBS, and centrifuged. FITC-*Cp*A-M5 in the supernatant was quantified fluorometrically (488 nm).

#### *2.13.4* *In vivo* distribution

The *in vivo* distribution of *Cp*A-M5 was tracked using fluorescence imaging. Mice were randomly assigned to two groups (n = 3): the microneedle group received *Cp*A-M5-MNs applied for 5 min, and the injection group received a subcutaneous injection of dissolved *Cp*A-M5-MN tip solution (20 μL). Whole-body fluorescence images were acquired at 0, 20, 40, and 60 min post-administration (excitation/emission: 488/525 nm; exposure: 500 m s).

### 2.14 Biosafety evaluation of *Cp*A-M5-MNs

#### 2.14.1 Hemocompatibility evaluation

*In vitro* hemolysis assay was performed to evaluate the hemocompatibility of each component of *Cp*A-M5-MNs. HA (15% w/v), *Cp*A-M5 (1 mg/mL), and PVA (10% w/v) were separately mixed and incubated for 30 min. Anticoagulated bovine blood was diluted with PBS, centrifuged, and washed until the supernatant was colorless, then resuspended to prepare a 5% red blood cell suspension. After incubation, the hemolysis rate was determined spectrophotometrically, with PBS and deionized water serving as the negative and positive controls, respectively.

#### 2.14.2 Cytocompatibility evaluation

HA, *Cp*A-M5, and PVA were prepared in PBS and added to cell culture wells. After 24 h of incubation, cell viability was determined using the CCK-8 method, and cell morphology was observed under a bright-field microscope.

#### 2.14.3 Histopathological examination

Histopathological effects of *Cp*A-M5-MNs on major organs in TCF-exposed mice were evaluated by H&E staining. Tissue sections from key organs were examined for morphological alterations to assess treatment-related toxicity.

### *2.15* *In vivo* therapeutic efficacy in TCF-induced poisoning mice

Twenty-four healthy male ICR mice (6–8 weeks old, 18 - 22 g) were obtained from Hangzhou Ziyuan Experimental Animal Technology Co., Ltd. (License No. SCXK (Zhejiang) 2019-0004) and acclimatized for one week under specific pathogen-free (SPF) conditions (25 ± 2 °C, 50 ± 10% humidity, 12 h light/dark cycle) with free access to food and water. Skin toxicity assessment was conducted in line with OECD guidelines (protocol 404, OECD, 2015) [31]. Mice were used, and TCF at different doses (5 mg/kg, 25 mg/kg, 50 mg/kg) was applied to the shaved skin areas, with 4-h exposure and 14-day continuous monitoring. Erythema and edema were scored based on Draize [32] and OECD criteria (0 - 4), and the Primary Irritation Index (PII) was categorized by irritation severity. Hair regrowth was evaluated in 4 stages [33]. All toxic signs and scoring data were recorded to assess TCF-induced dermal toxicity. All animal procedures were approved by the Animal Ethics Committee of Nanjing University (Approval No. IACUC-D2103016) and conducted in accordance with the Guide for the Care and Use of Laboratory Animals. Mice were randomly assigned to three groups (n = 8) [34]:

Control group: The shaved dorsal skin was cleansed with normal saline without further treatment.

Model group: After shaving and cleansing, 75 mg/kg bw of TCF (1/10 LD_50_) was topically administered to the skin in three consecutive applications (10 μL of 50 mg/mL TCF in methanol per application). The application site was covered with polyethylene film for 10 min after each administration to enhance penetration, and then rinsed off with saline after the final application. Acute toxic responses (erythema, edema) were monitored.

Treated group: Immediately after TCF exposure and film removal, a single *Cp*A-M5-MN patch was applied to the exposed area, pressed firmly for 5 min to ensure insertion, and then removed; the skin was gently cleansed with saline.

At 24 h post-treatment, all mice were euthanized by cervical dislocation. Full-thickness dorsal skin samples were collected: one portion was fixed in 4% paraformaldehyde, embedded in paraffin, sectioned, and stained with Masson's trichrome for histopathological evaluation. The remainder was snap-frozen in liquid nitrogen and stored at −80 °C for enzymatic and biochemical assays.

### 2.16 Tissue biochemical analysis

Histomorphological alterations in skin and major organs from TCF-exposed mice were evaluated by H&E and Masson's trichrome staining. Bright-field images were acquired at 40×, 100×, and 200× magnification to assess epidermal thickness, dermal inflammatory cell infiltration, collagen deposition, and visceral tissue architecture.

Cholinesterase (ChE) activity in skin, brain, liver, and kidney tissues was determined using the Ellman method. Frozen tissues were thawed on ice, and 0.1 g of each sample was homogenized in 1 mL of ice-cold PBS. The homogenate was centrifuged at 8000 rpm for 10 min at 4 °C, and the resulting supernatant was used for enzymatic analysis. Total protein concentration was quantified with a Bradford assay kit to normalize ChE activity. Serum ChE activity was measured directly by tracking absorbance at 412 nm using the Ellman method.

A panel of biochemical markers associated with skin inflammation and oxidative stress, as well as hepatic and renal function, was analyzed using commercial ELISA and colorimetric kits according to the manufacturers' protocols. Key biomarkers included pro-inflammatory cytokines (IL-6, IL-1β, IFN-γ, TNF-α), immunoglobulins (IgA, IgG), and oxidative stress parameters (T-AOC, SOD, CAT, MDA) in skin. Liver function was assessed by measuring ALT and AST levels, while renal function was evaluated based on blood BUN and CRE concentrations.

### 2.17 ^31^P- nuclear magnetic resonance (^31^P NMR) detection

SPE combined with ^31^P NMR spectroscopy was employed to detect TCF and its metabolites in skin tissues after *Cp*A-M5-MN treatment. Dorsal skin tissues (≈1.0 g) from the application site were excised, minced, and subjected to ultrasonic extraction with acetonitrile at 40 °C for 30 min. After centrifugation at 8000 rpm for 5 min, the supernatant was collected. Target analytes were eluted with acetonitrile, dried under a nitrogen stream, and prepared for NMR analysis.

^31^P -NMR spectra were acquired on a BRUKER AVANCE III 400 MHz NMR spectrometer [35]. Samples were re-dissolved in D_2_O, with TCF and DDVP standard solutions as internal standards and 85% orthophosphoric acid as the external standard. Spectral acquisition parameters were set as follows: resonance frequency 161.98 MHz, number of scans 32, pulse width 15.0000 μs, relaxation delay 2.0000 s, acquisition time 0.5112 s, and temperature 298.0 K. The characteristic phosphorus signal of TCF was identified at δ-18.3 ppm (P=O bond), and that of DDVP at δ-3.1 ppm (P-O-CH_3_ group). Spectral data were processed and visualized using MestReNova software.

### 2.18 Untargeted UHPLC-QTOF/MS metabolomics

Dorsal skin tissues (≈10 mg) were rapidly minced on dry ice and homogenized in 200 μL of ultrapure water using a high-throughput tissue homogenizer. Metabolites were extracted by adding 800 μL of pre-cooled methanol/acetonitrile (1:1, v/v), followed by vortexing for 1 min and incubation on ice for 30 min. After centrifugation (8000 rpm, 4 °C, 15 min), the supernatant was collected, vacuum-dried, and reconstituted in 100 μL of acetonitrile/water (1:1, v/v). The solution was filtered through a 0.22 μm nylon membrane prior to analysis. Quality control (QC) samples were prepared by pooling equal aliquots (10 μL) from all experimental samples and injected after every four sample runs to monitor instrument stability.

Metabolomic profiling was performed using an ultra-high-performance liquid chromatography-quadrupole time-of-flight mass spectrometry (UHPLC-QTOF/MS) system (Agilent 1290 Infinity LC coupled to an AB Sciex TripleTOF 6600). Chromatographic separation was carried out on an ACQUITY UPLC BEH C18 column (2.1 × 100 mm, 1.7 μm; Waters) maintained at 40 °C. The mobile phase consisted of 0.1% ammonium formate in water (solvent A) and acetonitrile (solvent B), with the following gradient: 0–1 min, 5% B; 1–12 min, 5–95% B; 12–14 min, 95% B; 14.1–16 min, 5% B. The flow rate was 0.3 mL/min, and the injection volume was 5 μL.

Mass spectrometry was conducted in both positive and negative electrospray ionization (ESI) modes under the following conditions: ion source temperature, 600 °C; spray voltage, ±5500 V; Gas1 and Gas2, 60 psi each; curtain gas, 35 psi; collision energy, 35 eV (±15 eV). Full-scan and MS/MS scans were acquired over *m*/*z* 60–1000 and 25–1000, respectively.

Raw data were processed using Progenesis QI for baseline correction, peak detection, retention time alignment, and normalization. Metabolic features with a relative standard deviation (RSD) > 30% in QC samples were excluded. Metabolite identification was performed by matching accurate mass (error <5 ppm), MS/MS spectra, and retention time against HMDB, KEGG, and METLIN databases, with further confirmation using authentic standards (identification level ≥2).

Differential metabolites were selected based on a variable importance in projection (VIP) > 1 from OPLS-DA and *p* < 0.05 from Student's t-test. Enriched metabolic pathways were identified using MetaboAnalyst 5.0 (Fisher's exact test, *p* < 0.05), and key pathways were visualized *via* Cytoscape 3.9.0 using weighted gene co-expression network analysis (WGCNA).

### 2.19 Statistical analysis

The data are presented as the mean ± standard error of the mean (SEM) from at least three independent experiments. Data analysis was performed using GraphPad Prism software 8.0 (San Diego, CA, USA). One-way analysis of variance (ANOVA) followed by Tukey's test was used to evaluate differences between datasets. *P* < 0.05 was considered statistically significant.

## 3 Results and discussion

### 3.1 Protective efficacy of *Cp*A-M5 in TCF-injured RAW 264.7 and HT-22 cells

**Fig. 2:**
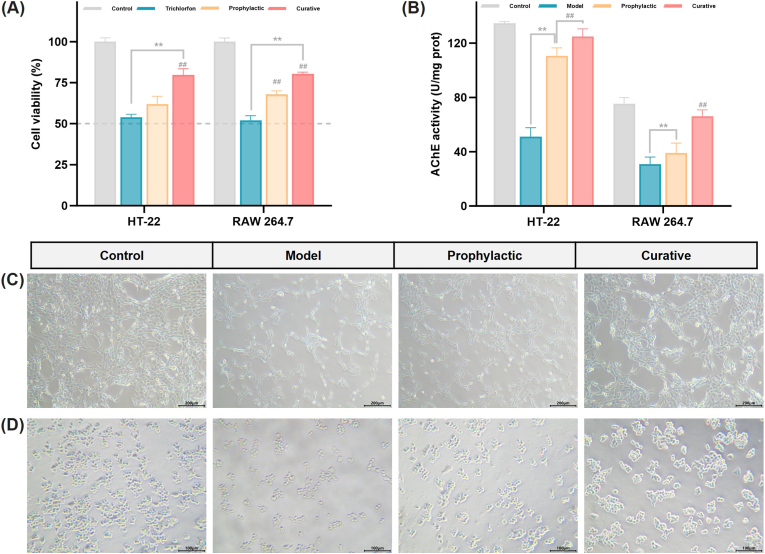
*Cp*A-M5 modulates the recovery of RAW 264.7 and HT-22 cells damaged by TCF. (A) Effect of *Cp*A-M5 prophylactic and curative treatments on the viability of TCF-damaged cells. (B) Analysis of cellular AChE activity. Bright-field microscopy images of (C) RAW 264.7 and (D) HT-22 cells.

In our previous work, the ability of *Cp*A-M5 to alleviate TCF-induced toxicity was evaluated using NIH/3T3 cells, a well-established model for skin barrier injury. As shown in the kinetic characterization (Table S1), *Cp*A-M5 exhibited improved catalytic efficiency (*k*_*cat*_/*K*_*m*_) compared with the wild-type enzyme, along with an appropriate substrate affinity toward acetylthiocholine, supporting its potential to act as an effective bioscavenger against organophosphate poisoning. On the basis of these validated enzymatic properties, RAW 264.7 and HT-22 cell models were further employed to systematically assess the protective effects of the mutant enzyme *Cp*A-M5 on pesticide-exposed cells, which mimics the immunoinflammatory microenvironment and neurotoxic milieu in skin tissues. The favorable biocompatibility of the exogenous enzyme *Cp*A-M5, as well as the cytotoxicity of TCF at different multiples of the acceptable daily intake (ADI), were determined using the CCK-8 assay (Fig. S1). Based on these preliminary data, exposure to TCF at a concentration of 10^2^ ADI (777 nM) for 16 h was selected as the cellular injury model for subsequent experiments (Fig. S1 C&D). Two intervention strategies were applied in this study, corresponding to prophylactic treatment (pre-incubation with M5 protein prior to TCF exposure) and curative treatment (treatment with M5 for 12 h post-TCF exposure), respectively. The effects of both treatments on TCF-impaired cell viability and AChE activity were investigated (Fig. 2). The results demonstrated that prophylactic treatment with M5 significantly restored cell viability and AChE activity, while curative treatment exerted a more pronounced protective effect against TCF-induced cellular damage.

This distinct efficacy difference between prophylactic and curative administration can be attributed to the unique toxicokinetic characteristics of low-volatile organophosphorus compounds and the extracellular working mechanism of exogenous *Cp*A-M5. Low-volatile OPPs quickly penetrate the skin to form a long-lasting dermal depot, from which toxins are slowly released into the extracellular space. In the prophylactic group, pre-incubated *Cp*A-M5 undergoes slow self-consumption before TCF exposure, leaving only low-activity enzyme that is gradually inhibited and unable to fully neutralize the toxin. In the curative group, by contrast, *Cp*A-M5 is added after TCF exposure and acts exclusively in the extracellular space without entering cells, allowing it to rapidly neutralize concentrated extracellular TCF and maximize its detoxification effect. These findings align well with the “catch-up therapy” concept, which highlights that post-exposure intervention targeting extracellular and skin-depot toxins yields more efficient detoxification than pre-exposure prophylaxis [24].

In real-world exposure scenarios, curative administration is far more clinically practical than prophylactic treatment, as percutaneous OPPs poisoning often remains unrecognized until delayed systemic symptoms appear [36,37]. Thus, the stronger protective effect of curative *Cp*A-M5 not only reflects its kinetic properties but also meets the critical clinical need for feasible countermeasures against real-world OPPs dermal poisoning.

### 3.2 Exogenous *Cp*A-M5 alleviates TCF-induced ROS accumulation and inflammatory responses

**Fig. 3:**
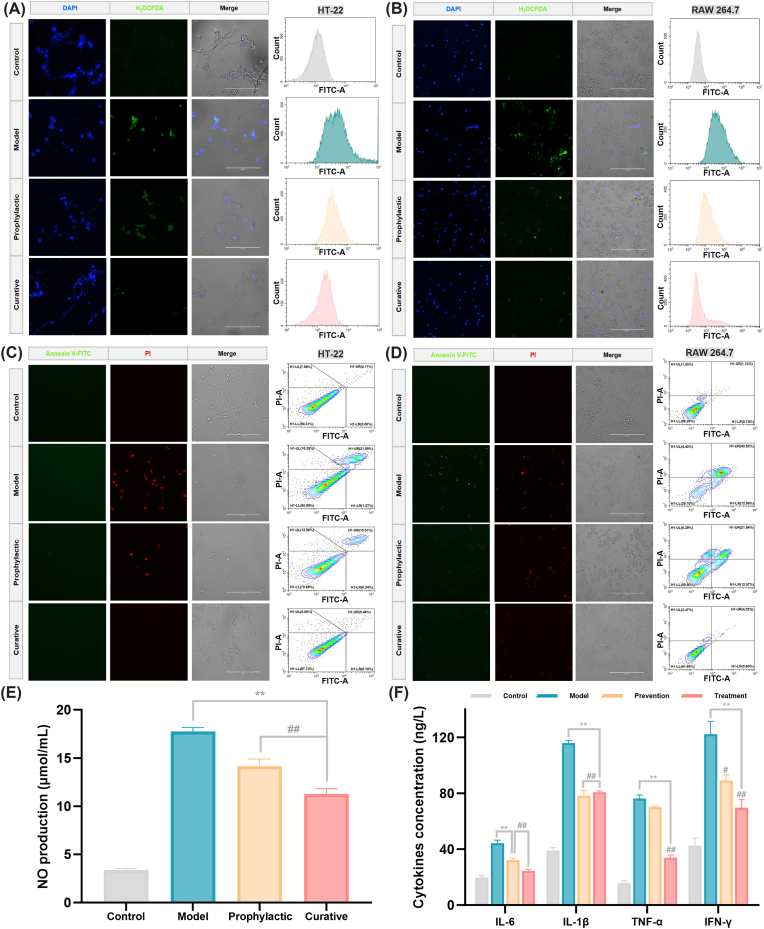
Exogenous *Cp*A-M5 reduces intracellular ROS levels and inhibits apoptosis by ameliorating TCF-mediated oxidative stress and inflammatory damage. Intracellular ROS levels in (A) HT-22 and (B) RAW 264.7 cells were assessed *via* H_2_DCFDA fluorescence staining combined with flow cytometry (scale bar = 200 μm). Apoptotic profiles of (C) HT-22 and (D) RAW 264.7 cells were analyzed using Annexin V-FITC/PI double fluorescence staining and flow cytometry (scale bar = 200 μm). Effects of M5 on the production of (E) NO and (F) inflammatory cytokines in cells were determined. Compared with the control group, ∗*p* < 0.05, ∗∗*p* < 0.01. Compared with the model group, ^#^*p* < 0.05, ^##^*p* < 0.01.

Numerous cellular studies have demonstrated that pesticide exposure elevates intracellular ROS levels and induces alterations in immune responses, which in turn disrupt cellular metabolism and trigger apoptosis. To this end, we measured the levels of ROS and inflammatory mediators to evaluate the protective effects of M5 on cells. As visualized by H_2_DCFDA fluorescence staining, TCF exposure remarkably elevated intracellular ROS levels in both HT-22 and RAW 264.7 cells (Fig. 3A and B). Prophylactic treatment with M5 effectively reduced ROS accumulation, while curative intervention post-TCF exposure exerted a more pronounced protective effect. We further investigated the impact of M5 on cell apoptosis (Fig. 3C and D). As illustrated in the figures, TCF treatment led to a significant increase in early and late apoptotic cells, characterized by green or red fluorescence signals. Notably, fewer early and late apoptotic cells were detected in the M5 curative treatment group. Additionally, the anti-inflammatory efficacy of M5 was assessed by measuring NO and inflammatory cytokine levels. The results showed that M5 treatment resulted in a significant reduction in these inflammatory indicators compared with the model group, with the curative group exhibiting statistically significant differences (Fig. 3E and F). It is well known that OPPs can induce not only cholinergic toxicity but also severe oxidative stress by impairing mitochondrial function and suppressing antioxidant defense systems [38]. Excessive ROS accumulation further acts as a central upstream signal that amplifies neuroinflammation, disturbs autophagic homeostasis, activates NF-κB signaling, and ultimately promotes apoptotic cell death [39]. As a potential underlying mechanism, *Cp*A-M5 may exert its protective effects by neutralizing TCF, thereby preventing the inhibition of endogenous antioxidant enzymes and interrupting the pathological cascade of oxidative stress, inflammation, and apoptosis.

### 3.3 The interaction mechanism of TCF/DDVP with *Cp*A-M5

**Fig. 4:**
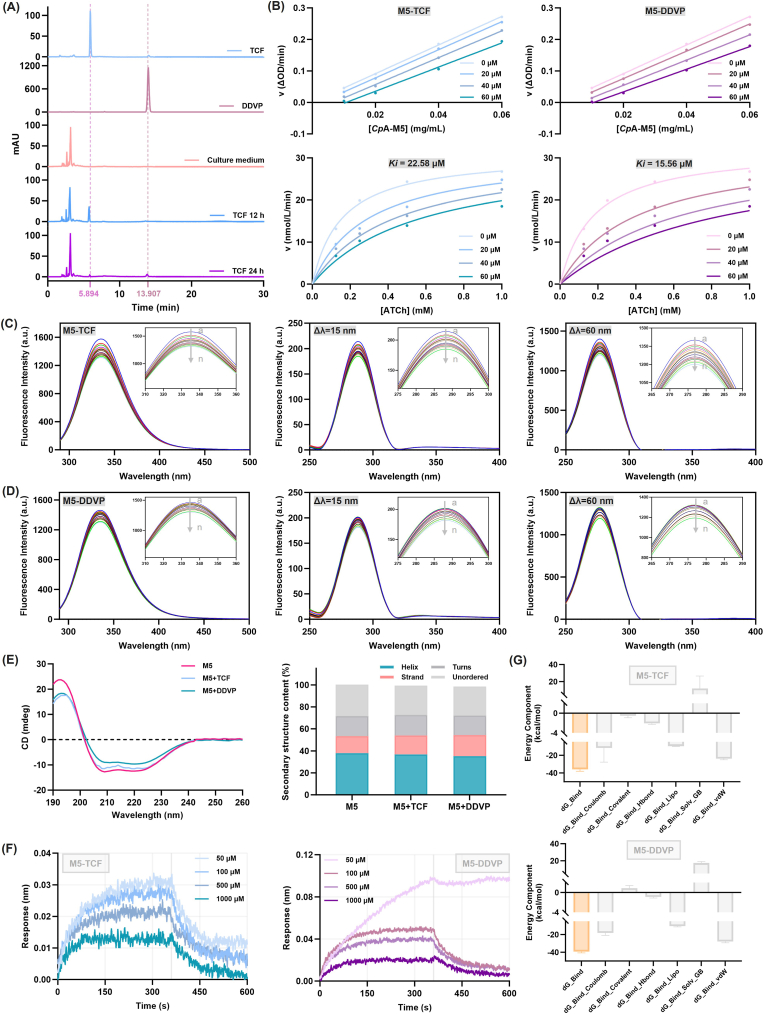
Enzyme inhibition and molecular interaction of TCF/DDVP with *Cp*A-M5. (A) HPLC analysis for the detection of TCF and its metabolite, DDVP, in cell culture medium. (B) The effects of different concentrations of TCF and DDVP on *Cp*A-M5 activity and substrate reaction kinetics. Concentration-dependent fluorescence quenching of *Cp*A-M5 by (C) TCF and (D) DDVP. (E) CD spectra and secondary structure content of *Cp*A-M5 after incubation with TCF and DDVP. (F) BLI method for measuring the binding affinity of TCF/DDVP to *Cp*A-M5. (G) MM-GBSA contribution of *Cp*A-M5-TCF and *Cp*A-M5-DDVP complex.

Considering the well-documented toxicity of TCF and its intrinsic instability in the environment that enables degradation into DDVP, HPLC was employed to determine whether DDVP was generated from TCF over a time course in the cell culture medium. The results revealed that TCF underwent time-dependent metabolic transformation in the cell culture medium (Fig. 4A). After 12 h of incubation, the characteristic chromatographic peak of DDVP was negligible in the medium. By 24 h, TCF degradation proceeded further, and a distinct DDVP peak became detectable. This transformation characteristic, featured by the coexistence of the parent compound and its metabolite in the environmental-biological system, may amplify the toxic effects of TCF under real-world exposure conditions. Therefore, it is critical to incorporate the monitoring of the metabolite DDVP into the pesticide toxicity assessment framework and further investigate the underlying mechanism of their interaction *in vitro*.

To investigate the inhibitory effects of TCF and its metabolite DDVP on *Cp*A-M5, we assessed their impacts on enzyme activity and substrate (ATCh) reaction kinetics. At fixed substrate concentrations, *Cp*A-M5 catalytic rate (v) decreased significantly with increasing TCF/DDVP concentrations (Fig. 4B). Parallel activity curves across inhibitor concentrations matched the kinetic signature of irreversible inhibitors. Nonlinear fitting yielded inhibition constants (*Ki*) of 22.58 μM (TCF) and 15.56 μM (DDVP), demonstrating DDVP's stronger binding affinity and inhibitory potency toward *Cp*A-M5, likely *via* covalent modification of the enzyme's active center essential groups to block catalysis.

Fluorescence and synchronous fluorescence spectroscopy analyses revealed concentration-dependent quenching of *Cp*A-M5 by TCF/DDVP (Fig. 4C and D). Free *Cp*A-M5 exhibited a characteristic emission peak at 340 nm, corresponding to Trp/Tyr hydrophobic microenvironments. Increasing TCF/DDVP concentrations reduced fluorescence intensity without peak shifts, indicating altered enzyme microenvironments *via* hydrophobic/hydrogen bonding; synchronous fluorescence further confirmed preferential perturbation of Trp-residue microenvironments with enhanced hydrophobicity. Stern-Volmer analysis (Table 1) showed DDVP's lower *K*_*sv*_ but 66-fold higher *K*_*a*_ than TCF, indicative of superior complex stability *via* specific binding. Both inhibitors exhibited *K*_*q*_ values far exceeding the dynamic quenching limit, confirming static complex formation. DDVP's near 1:1 binding stoichiometry (n = 0.940) and more negative *ΔG*° (−19.334 kJ/mol) indicated spontaneous, thermodynamically favorable binding, underpinning its stronger toxicological effect.

**Table 1:** The values of thermodynamic parameters for the binding of TCF or DDVP with *Cp*A-M5.

Compound	Stern-Volmer	*K*_*sv*_×10^4^ (M^−1^)	*R*^*2*^ (*K*_*sv*_)	*K*_*q*_×10^10^ (M^−1^ s^−1^)	*K*_*a*_ (M^−1^)	*n*	*R*^*2*^ (*K*_*q*_)	*ΔG°* (kJ/mol)
TCF	Y = 5916.1X+1.0631	5916.1	0.9898	59.161	37.145	0.492	0.9804	−8.956
DDVP	Y = 5044.8X+0.9975	5044.8	0.9686	50.448	2450.191	0.940	0.9686	−19.334

CD spectroscopy showed that untreated *Cp*A-M5 displayed typical α-helix negative peaks at 208/222 nm (Fig. 4E). TCF/DDVP incubation reduced peak intensity, indicating α-helix content loss, with concomitant fluctuations in random coil, β-sheet and β-turn proportions. DDVP's long alkyl chain may insert into *Cp*A-M5's hydrophobic pocket, disrupting α-helix-stabilizing hydrophobic stacking and inducing conformational changes to inhibit activity.

BLI revealed DDVP's lower equilibrium dissociation constant (*K*_*D*_ = 9.23 × 10^−6^ M *vs*. TCF *K*_*D*_ = 70.01 × 10^−6^ M) (Fig. 4F–Table 2), driven by slower dissociation rate (*k*_*dis*_), indicating more stable DDVP-*Cp*A-M5 complexes. Comparable association rates (*k*_*a*_) suggested initial hydrophobic interaction-driven binding for both inhibitors, elucidating DDVP's toxicity amplification as a TCF metabolite.

**Table 2:** Interaction parameters of TCF and DDVP with *Cp*A-M5.

Compound	*K*_*D*_×10^−6^ (M)	*k*_*a*_×10^2^ (M^−1^ s^−1^)	*k*_*dis*_×10^−4^ (s^−1^)
TCF	70.01 ± 1.20	1.087 ± 0.02	76.11 ± 0.37
DDVP	9.23 ± 0.33	1.049 ± 0.02	9.678 ± 0.28

MD simulations of *Cp*A-M5-TCF/DDVP complexes showed total binding free energies of −36.02 ± 2.13 kcal/mol (TCF) and −39.37 ± 1.48 kcal/mol (DDVP) (Fig. 4G, Fig. S2, Fig. S3). DDVP's stronger binding relied on enhanced Coulombic/van der Waals forces *via* multi-non-covalent interactions, consistent with experimental affinity/stability data. These results reveal the molecular basis of irreversible inhibition, supporting *Cp*A-M5-mediated cellular protection *via* TCF/DDVP binding and residual reduction.

### 3.4 Structure and performance characterization of *Cp*A-M5-MNs

**Fig. 5:**
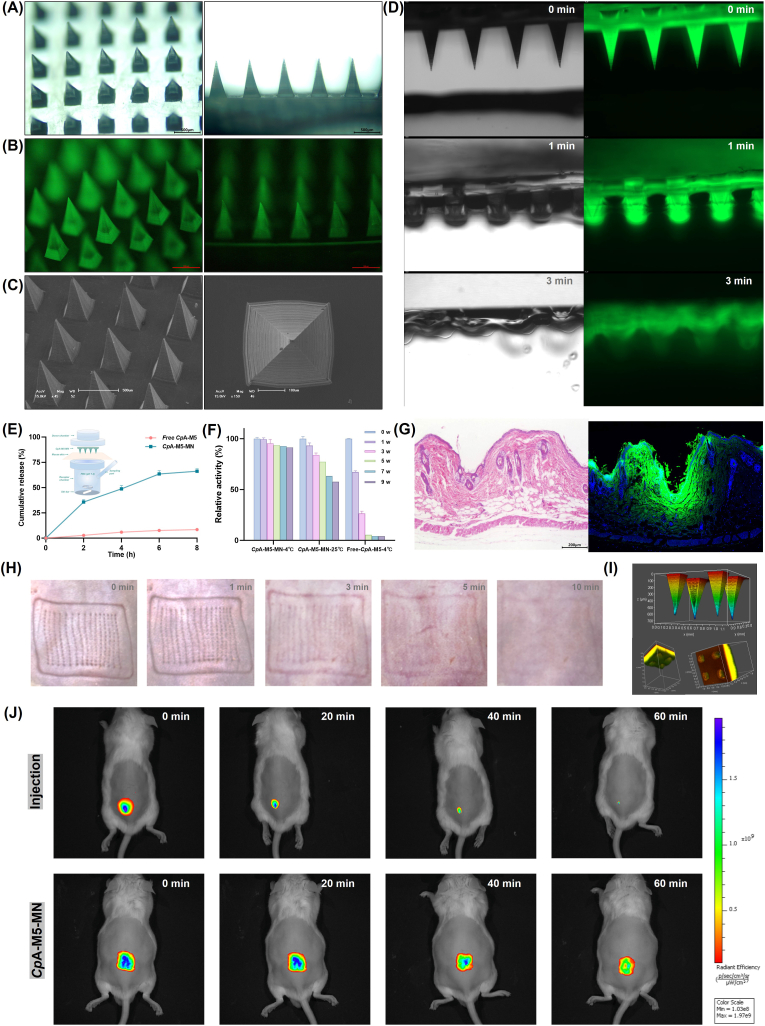
Structure and performance characterization of *Cp*A-M5-MN. (A) Optical microscope image of *Cp*A-M5-MN (×40). (B) Fluorescence microscopy images of *Cp*A-M5-MN (×40). (C) SEM image of *Cp*A-M5-MN. (D) Diffusion behavior of *Cp*A-M5-MN in 1% agarose gel, monitored *via* fluorescence imaging. (E) Cumulative release profile of *Cp*A-M5 from MNs in PBS buffer (pH 7.4, 37 °C). (F) Stability evaluation of *Cp*A-M5-MN stored at 4 °C and 25 °C. (G) H&E staining and fluorescence staining sections of mouse skin after insertion of *Cp*A-M5-MN. Green fluorescence: FITC-*Cp*A-M5, Blue fluorescence: DAPI-stained nuclei. (H) Recovery process of mouse skin after insertion of the microneedles. (I) 3D reconstruction image of *Cp*A-M5-MN and its dissolution in mouse skin after insertion, obtained by laser confocal microscopy. (J) Fluorescence imaging of mice at different time points (0, 20, 40, 60 min) after injection of FITC-*Cp*A-M5 or insertion of *Cp*A-M5-MN. (For interpretation of the references to color in this figure legend, the reader is referred to the Web version of this article.)

Following *in vitro* validation of *Cp*A-M5-mediated neutralization of TCF toxicity effect and its binding mechanism, a TCF percutaneous exposure model was established to evaluate the *in vivo* pharmacodynamics of *Cp*A-M5 *via* targeted enrichment at exposure sites using a MN system. FITC-labeled *Cp*A-M5 enabled fluorescence tracing (Fig. S4A), while optical/fluorescence microscopy and SEM were used to characterize MN morphological integrity and drug loading profiles. FT-IR analysis verified the absence of chemical interactions between the carrier and the loaded protein to preserve enzyme activity (Fig. S4B). As shown in Fig. 5A–C, *Cp*A-M5-MNs exhibited regular conical structures with sharp tips, uniform spacing and firm backing layer connection. FITC-*Cp*A-M5 was concentrated in tip centers, minimizing delivery wastage. Force-displacement curves confirmed MN structural integrity during puncture and eliminated the risk of needle breakage (Fig. S4C).

Free *Cp*A-M5, with its large molecular weight and hydrophilicity, is unable to effectively penetrate the skin barrier. In contrast, MNs physically pierce the stratum corneum and rapidly dissolve to deliver drugs directly into the dermis, overcoming the penetration limitations of conventional transdermal formulations. Fluorescence imaging results revealed that *Cp*A-M5-MNs dissolved rapidly within 1 min and achieved complete diffusion within 3 min in 1% agarose (Fig. 5D). Franz diffusion cell experiments were performed to evaluate the sustained-release property of *Cp*A-M5-MNs and their transdermal delivery efficiency relative to free enzyme, showing that the cumulative release rate of *Cp*A-M5-MNs was significantly higher than that of free *Cp*A-M5 (Fig. 5E). Solid-state storage of MN-loaded *Cp*A-M5 reduced enzyme denaturation risk compared with liquid free enzyme (Fig. 5F). Based on a 9-week continuous stability study, nonlinear kinetic fitting was performed to determine the half-life of *Cp*A-M5, revealing a half-life of free *Cp*A-M5 exhibited a half-life of only approximately 10 days under 4 °C storage, whereas 81 days for MN-loaded *Cp*A-M5 at room temperature.

H&E staining and fluorescence colocalization verified the transdermal efficiency. MNs generated ∼400 μm deep channels penetrating to the dermis, with FITC-*Cp*A-M5 diffusing uniformly along channels and colocalizing with DAPI-stained nuclei (Fig. 5G). CLSM 3D reconstruction visualized gradient drug diffusion from tips to dermis (Fig. 5I), confirming controlled release *via* HA matrix degradation.

*In vivo* safety and pharmacodynamic evaluations were carried out by assessing the hemocompatibility, cytotoxicity and immune response of MN materials (Fig. S4D–F), which indicated low risk of foreign body reactions. In mouse models, MN insertion only induced transient erythema that completely resolved within 10 min, verifying the minimally invasive nature of the MN system (Fig. 5H). *In vivo* fluorescence imaging showed that MN-delivered *Cp*A-M5 maintained high local fluorescence intensity for 60 min, whereas subcutaneous injection signals dissipated by 20 min (Fig. 5J), confirming prolonged target tissue retention that contributes to enhanced local detoxification efficiency. Although *Cp*A-M5 is a heterologous enzyme derived from insects, it represents a promising candidate for detoxification owing to its high catalytic efficiency and favorable stability. Notably, a variety of enzymes and proteins from bacterial, yeast, and mammalian sources have been approved for clinical use in humans [[40], [41], [42], [43], [44], [45]], with their immunogenicity and safety appropriately managed through strategies including PEGylation, high purity, local delivery, short-term administration, or Fc domain removal, thus validating the feasibility of heterologous proteins in medical applications. Importantly, the microneedle-based system restricts *Cp*A-M5 mainly to the dermal layer with minimal systemic exposure, which helps mitigate safety concerns associated with non-human enzymes. Localized and short-term application further reduces the risk of sustained immune recognition or systemic adverse reactions, supporting the practical use of engineered non-human enzymes in emergency cutaneous detoxification. Collectively, these findings demonstrate that *Cp*A-M5-MNs possess favorable biocompatibility, structural stability, and targeted transdermal delivery capacity, which provides critical safety assurance for their application as skin patches in pesticide exposure first aid and lays a solid foundation for subsequent *in vivo* detoxification experiments.

### 3.5 TCF detoxification pharmacological evaluation of *Cp*A-M5-MNs

Toxicity scoring showed that TCF (25 mg/kg, 50 mg/kg) caused mild skin irritation in mice without lethality (Table S3). Some TCF-treated animals exhibited slight erythema and edema (Draize/OECD score = 1), corresponding to a slightly irritating PII ≤2. No mortality or severe systemic toxicity was observed in any TCF-treated group over 14 days. Hair regrowth in exposed areas was at partial -to- advanced growth stages (P3 - P4), indicating reversible skin damage [46,47]. We further evaluated the therapeutic efficacy of *Cp*A-M5-MNs against TCF-induced skin injury. H&E staining showed intact epidermal structure with regular granular/spinous layers and wavy dermo-epidermal junction in normal mice, whereas TCF-exposed model mice exhibited epidermal hyperplasia, dermal neutrophil infiltration (black arrows), and amorphous eosinophilic deposits (red arrows). From necrotic keratinocytes and fragmented extracellular matrix. *Cp*A-M5-MN treatment restored epidermal thickness and normalized stratum corneum morphology, resembling the normal group. Masson staining revealed tightly arranged collagen bundles in normal skin. TCF exposure caused collagen swelling and fragmentation (blue arrows), while *Cp*A-M5-MN treatment re-established orderly collagen networks, supporting multi-dimensional repair of pesticide-induced skin toxicity.

**Fig. 6:**
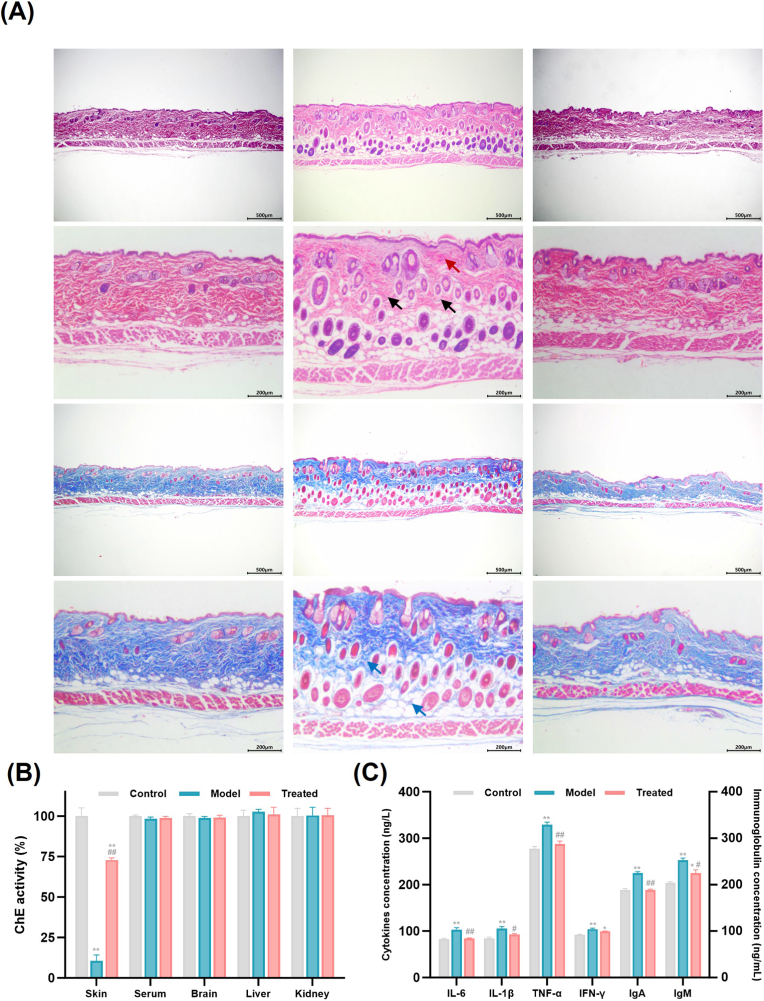
Histopathological and key biochemical evaluations of *Cp*A-M5-MNs against TCF-induced skin toxicity in mice. (A) Representative H&E-stained (×40, ×100) and Masson-stained skin sections (×40, ×100). (B) Relative activity of AChE in mouse skin, serum, brain, liver, and kidney tissues. (C) Cytokine and immunoglobulin levels in mouse skin.

The regulatory effect of *Cp*A-M5-MNs on TCF-induced toxic effects was systematically evaluated by detecting biochemical indicators in mouse organs. As shown in Fig. 6B, ChE activity in the skin tissue of the model group was significantly lower than that of the normal group, while ChE activity in serum, brain, liver, and kidneys remained normal. This suggests that TCF-induced toxicity is mainly localized to the skin exposure site, where it inhibits ChE activity and causes local cholinergic nerve dysfunction. After *Cp*A-M5-MN treatment, skin ChE activity was restored to 72% of the normal group, indicating that *Cp*A-M5 delivered by microneedles can specifically neutralize TCF and its metabolites in the skin, block their irreversible phosphorylation of endogenous ChE during acute toxicity, with low risk of systemic toxicity. Inflammation analysis (Fig. 6C) showed TCF-induced elevation of skin pro-inflammatory cytokines (IL-6, IL-1β, TNF-α, IFN-γ), consistent with Th2-type allergic dermatitis-like pathology reported in pesticide-exposed populations [48]. Excessive TNF-α may activate NF-κB signaling to exacerbate inflammation and vascular damage, while increased skin IgA/IgM levels implied humoral immune imbalance, similar to observations in pesticide-exposed fish [49]. *Cp*A-M5-MNs significantly reversed these inflammatory and immunotoxic changes.

As shown in Table 3, TCF exposure decreased skin T-AOC, CAT and SOD activities, leading to ROS accumulation and lipid peroxidation. This in turn attacks polyunsaturated fatty acids in cell membranes, generating lipid peroxidation end products such as MDA. Consistent with the well-documented toxicological mechanism of organophosphates, TCF-induced suppression of antioxidant enzymes (CAT, SOD) and reduction of T-AOC disrupt the balance between oxidative stress and antioxidant defense systems, further exacerbating ROS overproduction and lipid peroxidation [50]. Notably, *Cp*A-M5-MN treatment effectively reversed these pathological changes: restoring T-AOC and CAT to normal levels and partially recovering SOD activity, which directly alleviates oxidative damage by enhancing the cell's ability to scavenge excess ROS and inhibit lipid peroxidation. This protective effect is attributed to the ability of *Cp*A-M5 to neutralize TCF and its metabolite DDVP, thereby preventing further impairment of endogenous antioxidant enzymes and breaking the vicious cycle of oxidative stress amplification [51]. Histopathological examination of major organs (liver, kidney, thymus, spleen, Fig. S5, Table S4) showed no significant lesions across groups, confirming the safety of *Cp*A-M5-MN local detoxification strategy.

**Table 3:** The effect of *Cp*A-M5-MN on oxidative stress markers in the skin of TCF injured mice.

Group	T-AOC (μmol Trolox/mL)	CAT (U/mg prot)	SOD (U/mg prot)	MDA (nmol/mg prot)
Control	6.44 ± 0.17	56.47 ± 1.34	34.91 ± 1.41	18.04 ± 0.38
Model	5.19 ± 0.11∗∗	39.15 ± 0.87∗∗	22.58 ± 2.18∗∗	20.01 ± 0.55∗∗
Treated	6.46 ± 0.13^##^	55.67 ± 2.48^##^	25.41 ± 2.12∗∗	18.65 ± 0.37^#^

### 3.6 Effects of *cpa*-m5-mn on skin tissue metabolites in TCF-exposed mice

Prior detection of DDVP levels in cell culture media and investigation into small molecule-protein binding mechanisms suggested that TCF metabolites may induce skin toxicity. However, DDVP and other phosphorus-containing compounds were undetectable in skin samples *via* ^31^P NMR (Fig. S6), which could be attributed to rapid TCF metabolism, systemic absorption, or low extraction efficiency of low-abundance metabolites. Comprehensive elucidation of the detoxification mechanisms of *Cp*A-M5-MNs and the biosafety of metabolic end products requires further integration of untargeted metabolomics.

**Fig. 7:**
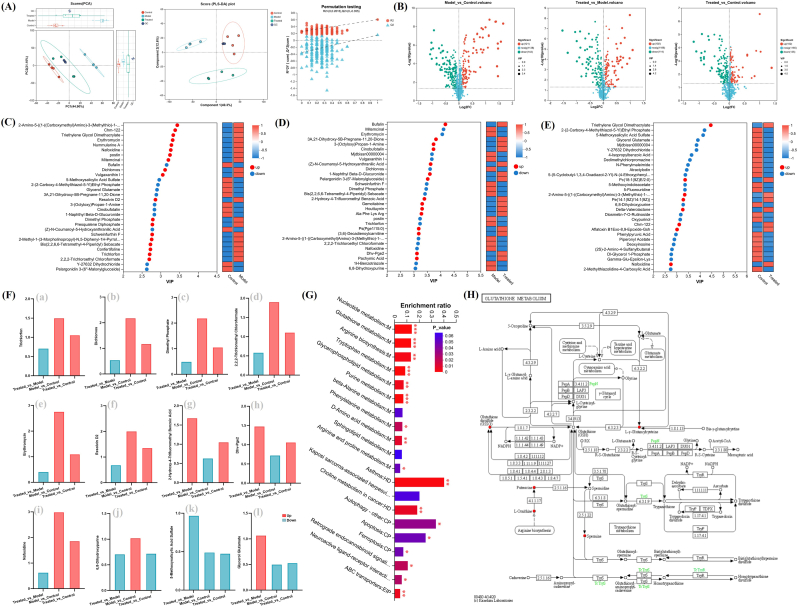
Differential metabolites and pathway analysis of *Cp*A-M5-MN intervention in TCF-exposed mouse skin. (A) Metabolic profiling pattern analysis of skin metabolites in Control, Model, and Treated groups of mice. (B) The volcano plot of differentially metabolites. The VIP plot of metabolites in multivariate statistical analysis. (C) Control *vs.* Model; (D) Mode *vs.* Treated; (E) Control *vs.* Treated. (F)Fold change of metabolite expression between the two groups. (G) KEGG enrichment analysis (∗*p* < 0.05, ∗∗*p* < 0.01, ∗∗∗*p* < 0.001). (H) Glutathione metabolism.

Principal component analysis (PCA) showed distinct spatial separation of skin metabolic profiles among normal, model, and *Cp*A-M5-MN-treated groups (Fig. 7A), confirming significant metabolic perturbations induced by TCF and reversal by CpA-M5-MN intervention. Partial least squares discriminant analysis (PLS-DA) with permutation tests (200 iterations) validated model reliability without overfitting, as evidenced by Q2 values below original data points and negative y-axis intercepts.

Differential metabolites were screened using VIP ≥1 (PLS-DA) and FC ≥ 2/≤ 0.5 (univariate analysis). Volcano plots (Fig. 7B) revealed 121 upregulated and 143 downregulated metabolites between normal and model groups, 102 upregulated and 114 downregulated between model and treatment groups, and 102 upregulated and 135 downregulated between normal and treatment groups. Top 30 VIP metabolites (Fig. 7C–E) were prioritized for functional analysis.

As shown in Fig. 7F(a-d), TCF as a prodrug and its downstream metabolites (DDVP, Dimethyl Phosphate, and 2,2,2-Trichloroethyl Chloroformate) were elevated in the model group, while *Cp*A-M5-MN treatment significantly reduced their levels, consistent with ^31^P NMR results and confirming disruption of the toxic metabolic chain. Fig. 7F(e-f) demonstrated that erythromycin and Resolvin D2 levels were elevated in the model group and reduced following *Cp*A-M5-MN treatment. As an antibiotic, erythromycin may alleviate inflammation indirectly by inhibiting bacterial infection; however, its upregulation in the model group implies the proliferation of drug-resistant bacteria or activation of immune compensatory mechanisms. Resolvin D2, a pro-resolving mediator of inflammation, showed increased abundance in the model group, which likely reflects a compensatory feedback response to impaired inflammatory resolution. The decreased levels of both metabolites in the treatment group indicated that *Cp*A-M5-MN-mediated targeted detoxification alleviated local inflammation by reducing the reliance on antibiotics and pro-resolving mediators.

Fig. 7F(g-h) showed that the levels of 2-Hydroxy-4-Trifluoromethyl Benzoic Acid, a trifluoromethylbenzoic acid derivative, and Dhv-Pge2, a prostaglandin analog, were decreased in the model group and restored in the treatment group. The benzoic acid derivative exerts anti-inflammatory effects potentially through ROS scavenging or cyclooxygenase-2 (COX-2) inhibition, and its reduction in the model group suggests a loss of control over inflammatory regulation. Dhv-Pge2, as a modified prostaglandin, may activate the prostaglandin E receptor 4 (EP4) to promote tissue repair, and its depletion in the model group exacerbates inflammatory damage. The recovery of these two metabolite levels in the treatment group verified that *Cp*A-M5-MN intervention restored the oxidative-inflammatory balance in damaged skin tissues.

Other differential metabolites showed distinct expression patterns across groups. Nafoxidine accumulated in the model group, over-inhibiting estrogen-mediated skin barrier repair and hair follicle metabolism, and exacerbating apoptosis and oxidative stress. 6,8-Dihydroxypurine, a purine oxidation product, was elevated in the model group, indicating purine metabolic disorder, aggravated oxidative stress, and subsequent DNA damage and inflammation amplification. Certain metabolites also revealed limitations of *Cp*A-M5-MN intervention. 5-Methoxysalicylic Acid Sulfate, a salicylic acid sulfated metabolite with ROS-scavenging and anti-inflammatory properties, remained decreased in both model and treatment groups, suggesting impaired sulfation pathways and persistent oxidative damage risk. Glycerol Glutamate, an osmotic/energy metabolism regulator, was elevated in the model group (reflecting cellular stress) but reduced below normal levels post-treatment, indicating that while *Cp*A-M5 alleviates stress, excessive reduction may disrupt water balance and hinder skin barrier repair.

KEGG pathway analysis identified 17 significantly enriched metabolic pathways (Fig. 7G), including glutathione metabolism, arginine biosynthesis, autophagy, and neuroimmune interaction, which formed a complex regulatory network (Fig. 7H). In the model group, glutathione metabolism was dysregulated with elevated oxidized glutathione and reduced γ-L-Glutamyl-L-Cysteine, while ferroptosis activation confirmed oxidative stress as the core toxicity mechanism. Arginine metabolism perturbation, featured by increased N2-Acetylornithine and decreased Citrulline and L-Ornithine, impaired antioxidant defenses. Reduced Putrescine and Deoxyinosine indicated autophagy inhibition and toxic accumulation. Neuroimmune pathways were activated, with elevated Histamine and abnormal Tryptophan/Purine metabolism amplifying inflammation and energy imbalance. *Cp*A-M5-MN treatment restored L-Ornithine, Spermine, and Deoxyinosine levels, partially reversing key metabolic perturbations.

Pearson correlation analysis was conducted to link metabolic biomarkers of *Cp*A-M5 intervention with serum biochemical parameters (Fig. S7), revealing strong negative correlations between metabolites like Pelargonidin 3-(6″-Malonylglucoside) and pro-inflammatory cytokines (TNF-α, IL-6) as well as oxidative stress markers (MDA, CAT), and positive correlations of 3-(Octyloxy)Propan-1-Amine and Oleoyl-L-Carnitine with skin AChE activity, antioxidant enzymes (CAT, T-AOC) while negatively correlating with IL-6 and MDA. Prednisolone Tebutate showed positive correlations with pro-inflammatory cytokines and negative correlations with antioxidant markers, collectively indicating that *Cp*A-M5 alleviates pathological damage by restoring key metabolic nodes in oxidative and amino acid metabolism pathways, consistent with previous metabolomic and pathway analysis.

Collectively, combining *in vivo* data and KEGG enrichment analysis reveals that TCF triggers skin damage *via* an interconnected network of glutathione metabolism, neuroactive ligand-receptor interaction, and amino acid metabolism pathways, causing membrane impairment, metabolic imbalance, and inflammation amplification. *Cp*A-M5-MN intervention partially rescues key metabolic nodes, providing a solid basis for targeted detoxification strategies.

## 4 Conclusions

In summary, this study developed a *Cp*A-M5-loaded microneedle system for the targeted detoxification of TCF-induced dermal toxicity and clarified the corresponding *in vitro* and *in vivo* detoxification efficacy and mechanisms. *Cp*A-M5 exerts protective effects on cells by binding to TCF, which prevents TCF from inhibiting endogenous ChE activity and mitigates the suppression of antioxidant defense systems (including SOD, CAT, and GSH-Px). This protective effect further scavenges excessive intracellular ROS, breaks the pathological cascade of oxidative stress amplification, inhibits neuroinflammation, and ultimately alleviates cell apoptosis. Enzyme kinetics and molecular interaction assays confirmed the irreversible binding of *Cp*A-M5 to TCF and DDVP, with DDVP exhibiting higher binding affinity and forming more stable complexes. *In vivo*, the microneedle system enabled the targeted delivery of *Cp*A-M5 to dermal sites, reducing residual TCF/DDVP levels, repairing skin barrier damage, restoring local oxidative-inflammatory homeostasis, and regulating the glutathione metabolic pathway. Several challenges and limitations must be noted. Specifically, the long-term *in vivo* immunogenicity of the *Cp*A-M5-MNs system, as well as its targeting precision for detoxification sites, remains to be further verified. Moreover, it is necessary to expand the scope of research to include more types of pesticides, which will help enhance the universality of the detoxification strategy and facilitate its potential clinical translation. Collectively, this work provides a novel exogenous enzyme delivery strategy for pesticide-induced dermal toxicity and lays a theoretical and experimental foundation for the clinical translation of engineered enzyme-based bioscavengers in dermal protection and emergency detoxification.

## CRediT authorship contribution statement

**Shuoqi Jiang:** Funding acquisition, Investigation, Validation, Writing – original draft. **Zi-Wei Zheng:** Methodology, Resources, Supervision. **Qiuya Gu:** Methodology, Resources. **Jian-Xin Li:** Funding acquisition, Methodology, Resources. **Xiaobin Yu:** Funding acquisition, Resources, Supervision. **Zhuangwei Zhang:** Funding acquisition, Investigation, Methodology, Writing – review & editing.

## Declaration of competing interest

The authors declare that they have no known competing financial interests or personal relationships that could have appeared to influence the work reported in this paper.

## Data Availability

Data will be made available on request.
