# Supplementary material for: Engineered acetylcholinesterase-loaded dissolvable microneedles mitigate dermal toxicity by targeting trichlorfon binding and metabolic pathway modulation
Source: Mater Today Bio. 2026 May 14;38:103243. doi: 10.1016/j.mtbio.2026.103243 (PMC13197772; doi:10.1016/j.mtbio.2026.103243)
Supplement: Multimedia component 1 [file mmc1.docx]

Supplementary Information

**Engineered Acetylcholinesterase-Loaded Dissolvable Microneedles Mitigate Dermal Toxicity by Targeting Trichlorfon Binding and Metabolic Pathway Modulation**

Shuoqi Jiang^1, 2^, Zi-Wei Zheng^1,^ *, Qiuya Gu^2^, Jian-Xin Li^4^, Xiaobin Yu^2,^ *, Zhuangwei Zhang^3, 4,^ *

*^1^Digital Industry Research Institute, Zhejiang Wanli University, No.8 South Qian Hu Road, Ningbo, Zhejiang, China*

*^2^**Key Laboratory of Industrial Biotechnology, Ministry of Education, School of Biotechnology, Jiangnan University, 1800 Li-Hu Road, Bin-Hu District, Wuxi, Jiangsu, China*

*^3^**Central Laboratory of the Medical Research Center, The First Affiliated Hospital of Ningbo University, Ningbo, Zhejiang, China*

*^4^State Key Laboratory of Analytical Chemistry for Life Science, Collaborative Innovation Centre of Chemistry for Life Sciences, Jiangsu Key Laboratory of Advanced Organic Materials, School of Chemistry and Chemical Engineering, Nanjing University, Nanjing, Jiangsu, China*

Shuoqi Jiang E-mail: [jsq_sxty@163.com](mailto:jsq_sxty@163.com)

Qiuya Gu E-mail: guqiuya@aliyun.com

Jian-Xin Li E-mail: lijxnju@nju.edu.cn

* Corresponding author: Zi-Wei Zheng, Xiaobin Yu, Zhuangwei Zhang

Corresponding author E-mail: [zhengziwei@tsinghua.org.cn](mailto:zhengziwei@tsinghua.org.cn) (Zi-Wei Zheng), [xbyu@jiangnan.edu.cn](mailto:xbyu@jiangnan.edu.cn) (Xiaobin Yu), fyzhangzhuangwei@nbu.edu.cn (Zhuangwei Zhang)

Tel. and Fax: +86 178 6797 0909


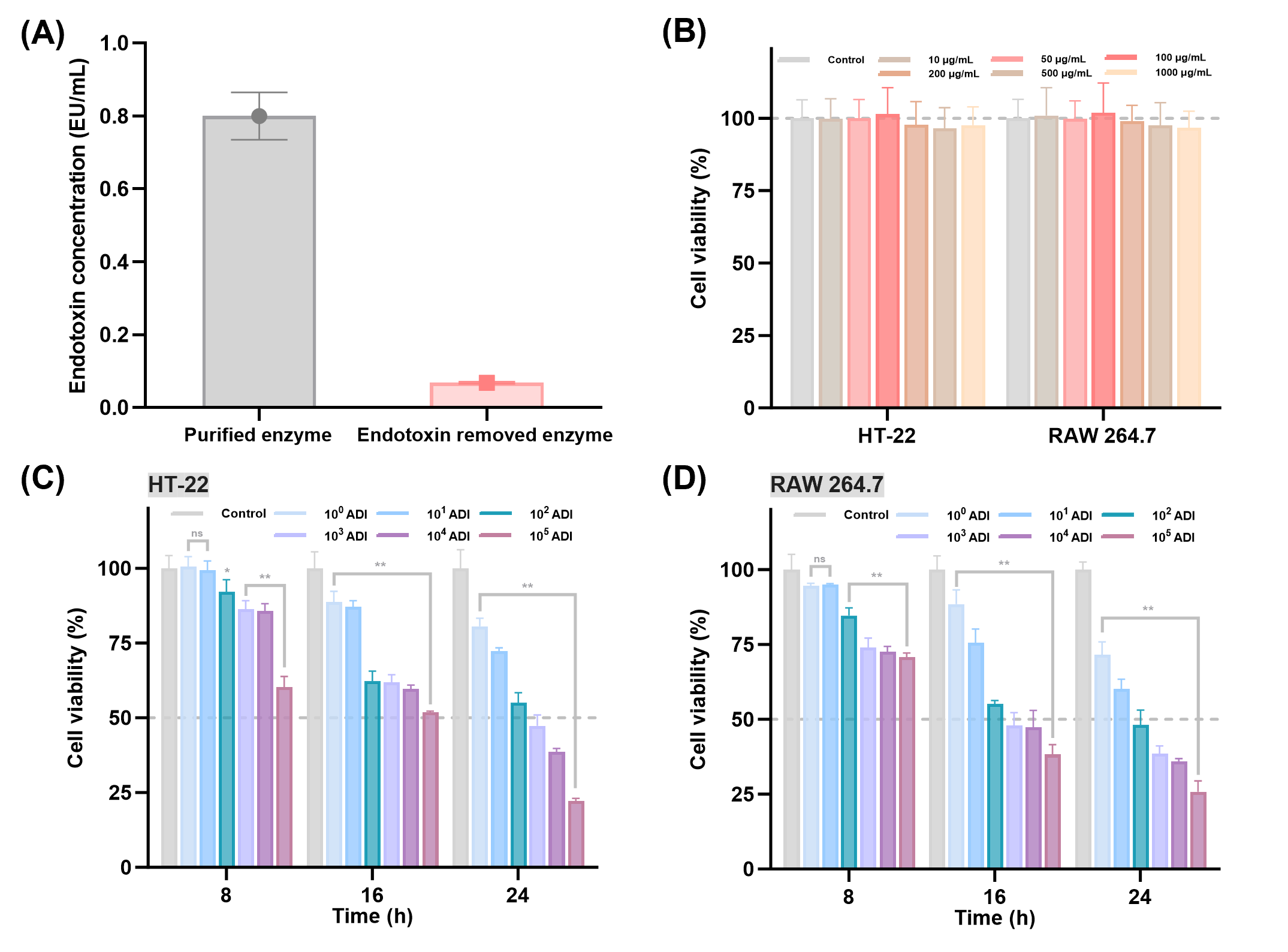


**Fig. S1.** Effects of *Cp*A-M5 and TCF on cell viability. (A) Endotoxin contents in purified enzyme and endotoxin removed enzyme. (B) The effects of *Cp*A-M5 on the viability of HT-22 and RAW 264.7 cells. The effects of TCF exposure on the viability of (C) HT-22, and (D) RAW 264.7 cells.


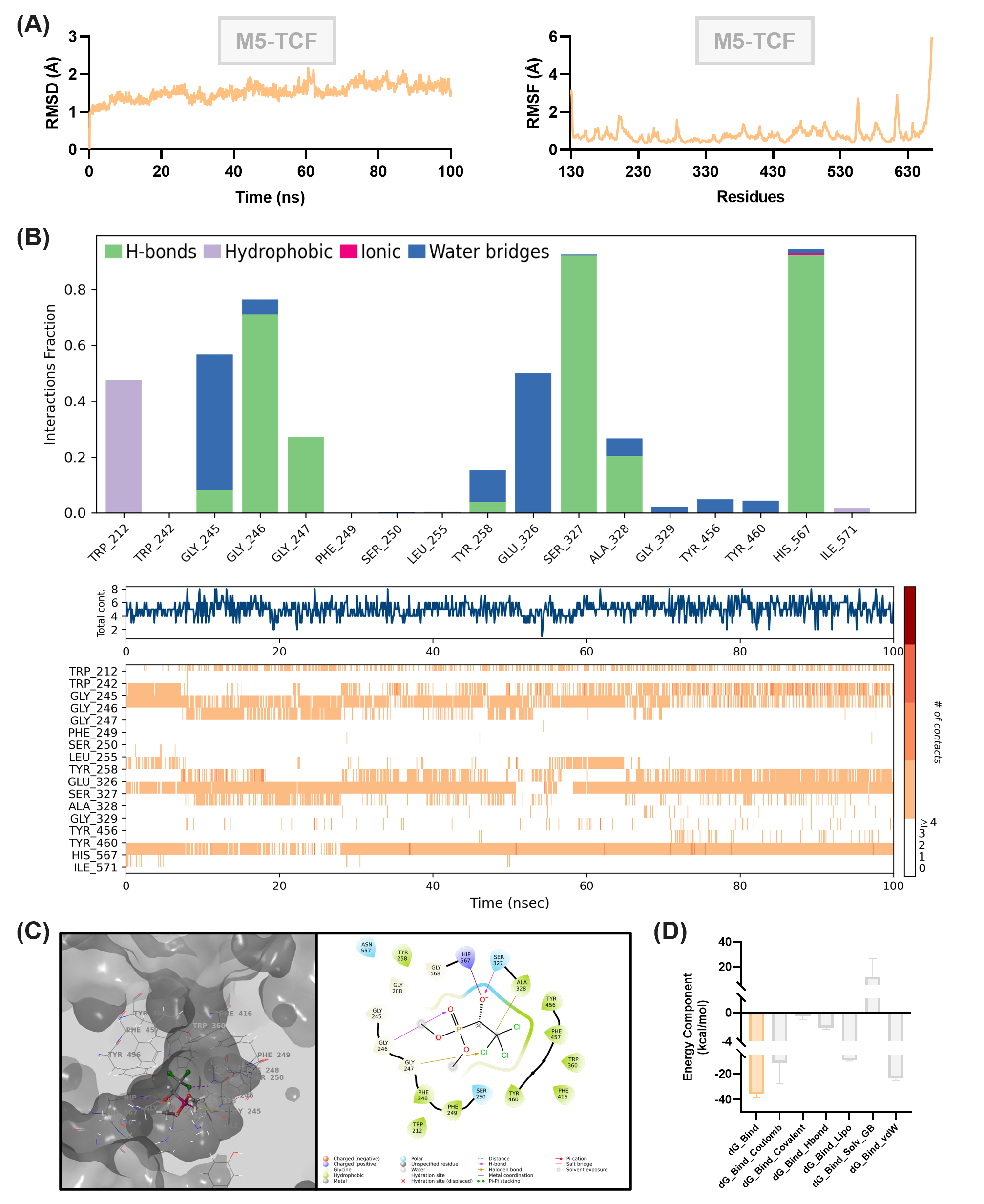


**Fig. S2.** MD simulation analysis of *Cp*A-M5 interactions with TCF. (A) RMSD and RMSF. (B) Various interaction types and the timeline representation of contacts throughout the simulation of *Cp*A-M5-TCF complex. (C) Schematic representation of TCF interactions with *Cp*A-M5. (D) MM/GBSA contribution of complex.

**
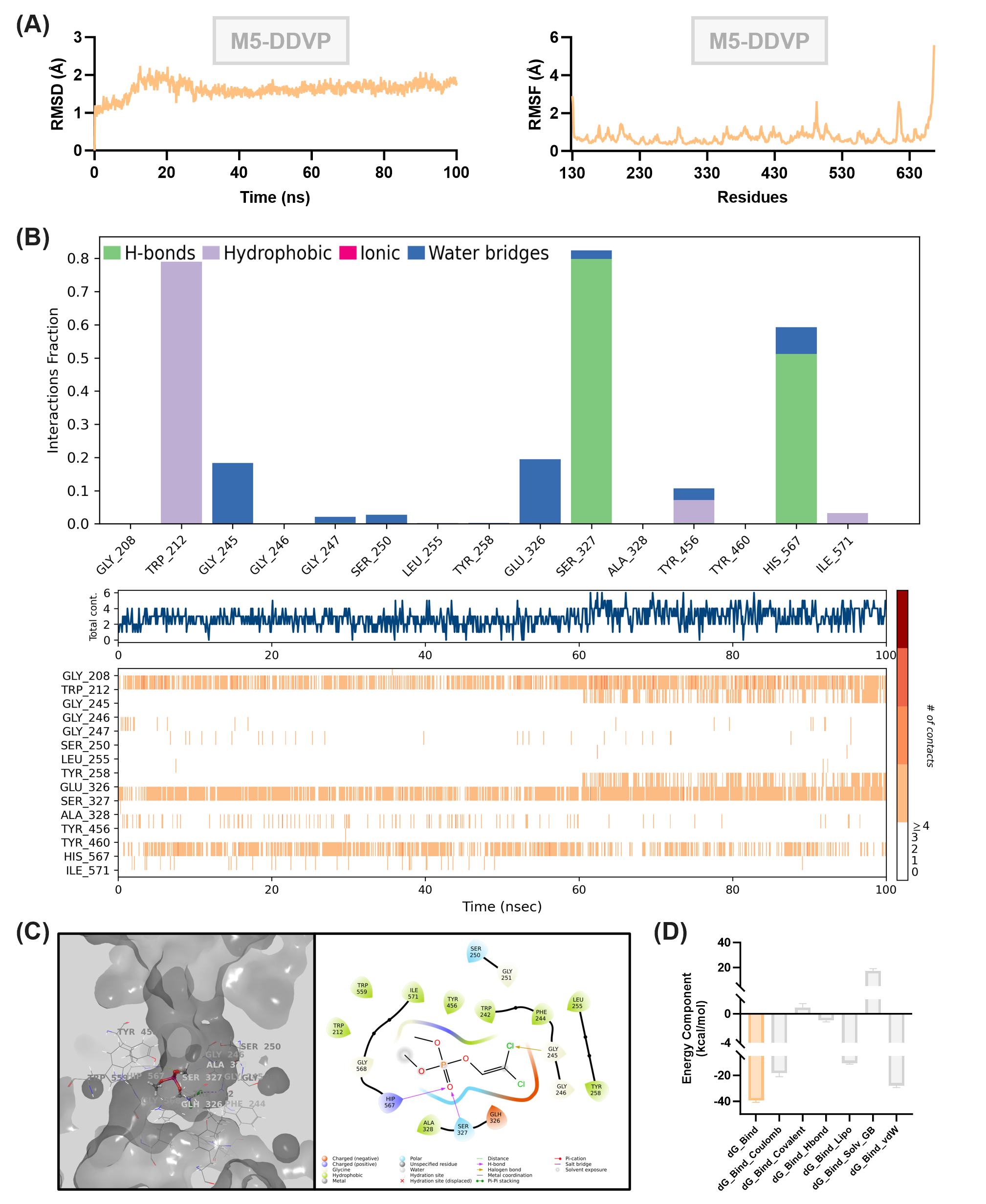
**

**Fig. S3.** MD simulation analysis of *Cp*A-M5 interactions with DDVP. (A) RMSD and RMSF. (B) Various interaction types and the timeline representation of contacts throughout the simulation of *Cp*A-M5-DDVP complex. (C) Schematic representation of DDVP interactions with *Cp*A-M5. (D) MM/GBSA contribution of complex.

**
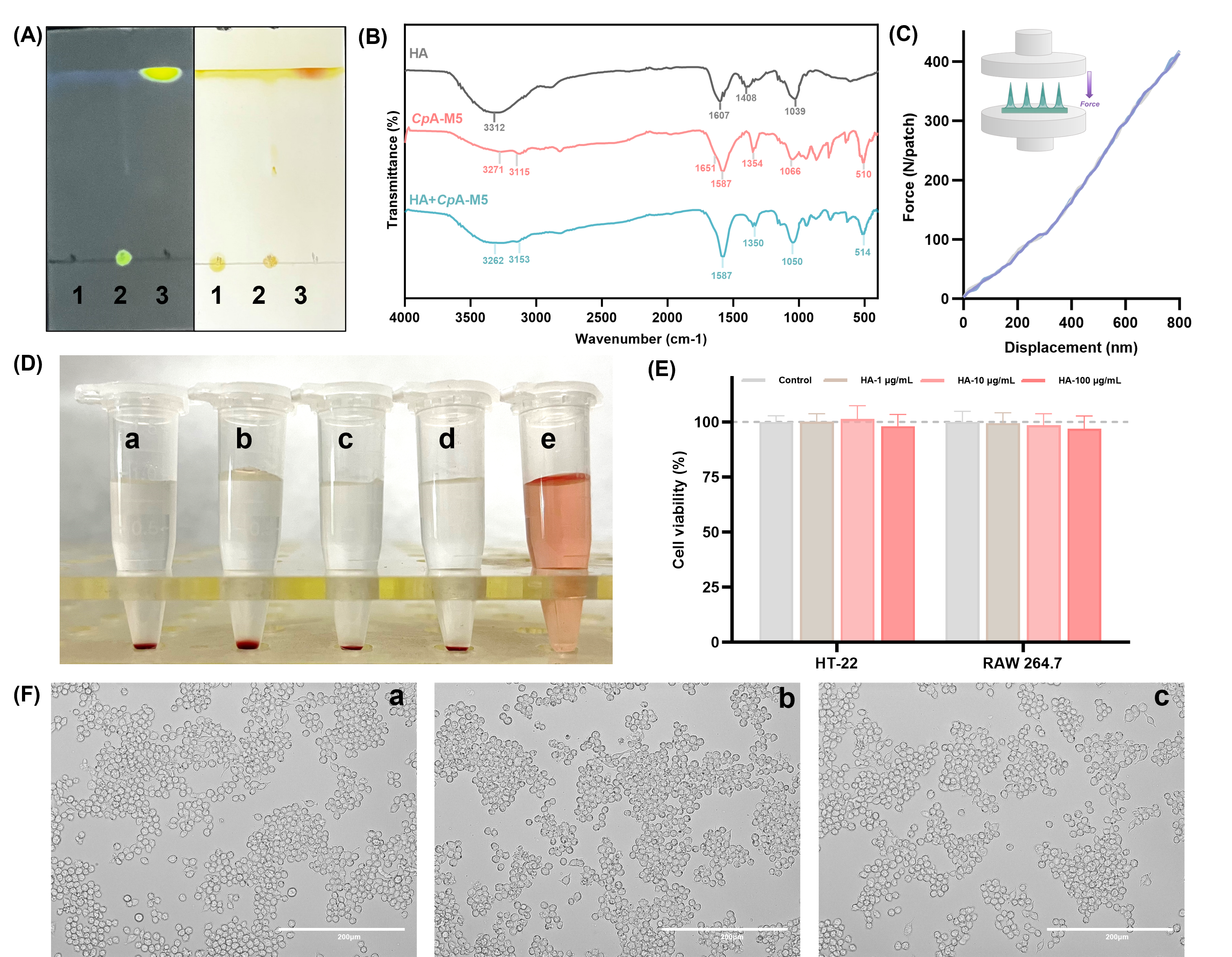
**

**Fig. S4.** Structure and performance characterization of *Cp*A-M5-MN. (A) Identification of FITC labeled *Cp*A-M5 by TLC method, application point 1: *Cp*A-M5, 2: FITC-*Cp*A-M5, 3: FITC. (B) FTIR spectra of HA, *Cp*A-M5, HA+*Cp*A-M5. (C) The force-displacement curves of *Cp*A-M5-MN and schematic illustration of the test setup. (D) Hemolysis assay of *Cp*A-M5-MN components, a: HA, b: *Cp*A-M5, c: PVA, d: Negative control (PBS), e: Positive control (deionized water). (E) Cytotoxicity of different concentrations of HA on HT-22 and RAW 264.7 cells. (F) Evaluation of pro-inflammatory potential of *Cp*A-M5-MN components in RAW 264.7 cells, a: Control，b: HA, c: PVA.

**
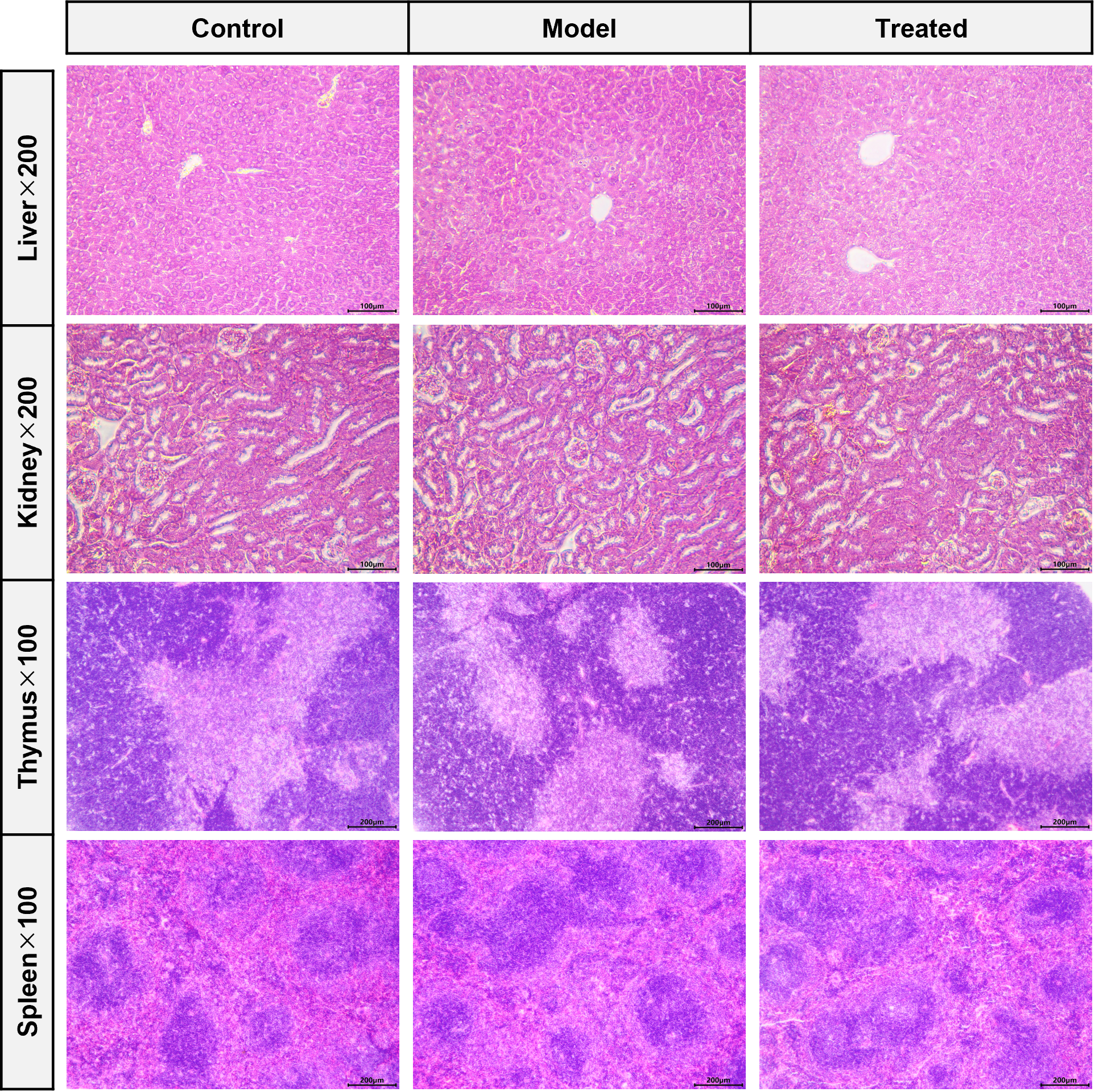
**

**Fig. S5.** Representative H&E-stained sections of: Liver (×200), Kidney (×200), Thymus (×100), and Spleen (×100).


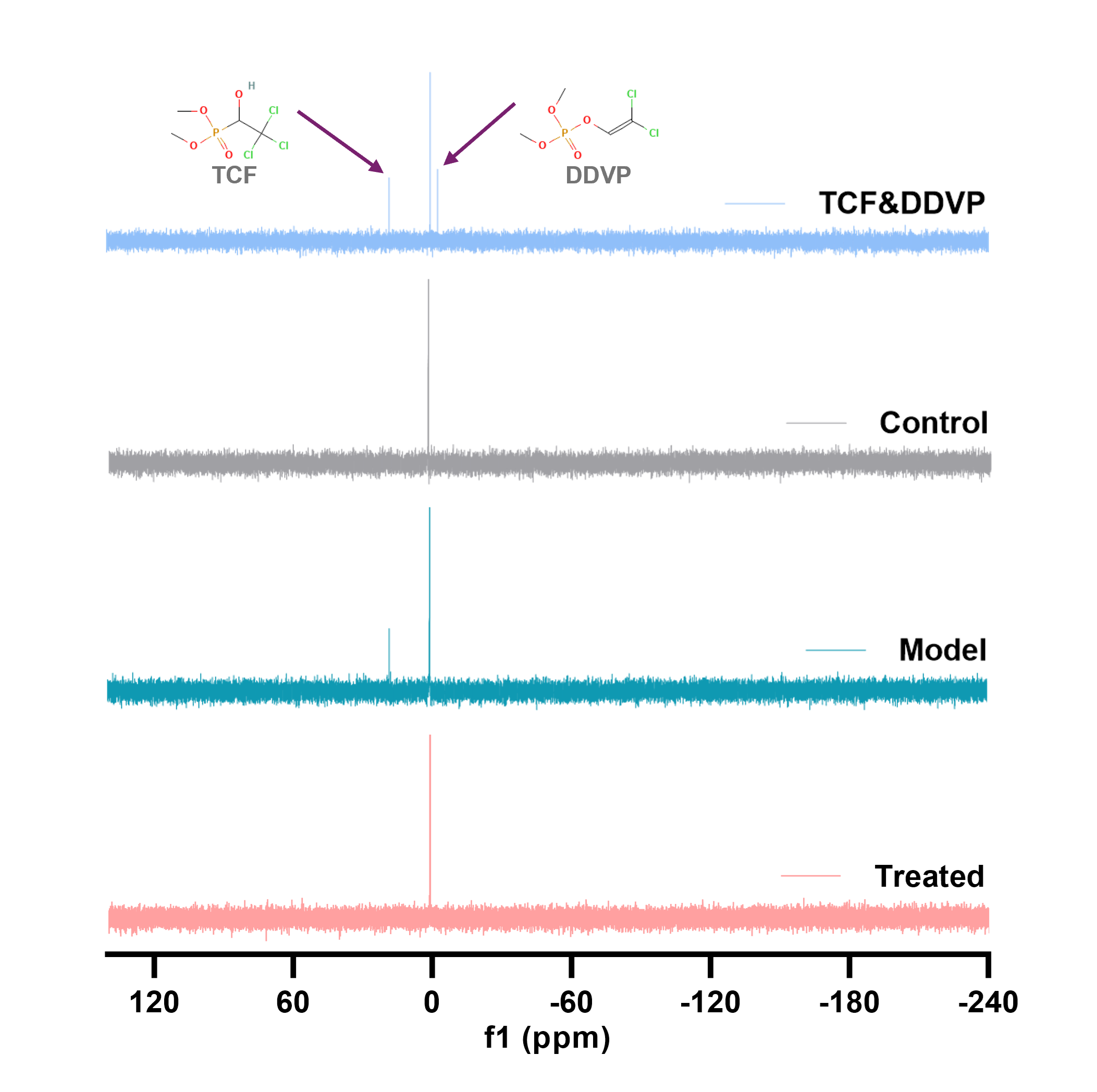


**Fig. S6.** ^31^P-NMR profiling of TCF, DDVP standards and TCF-treated mouse skin.


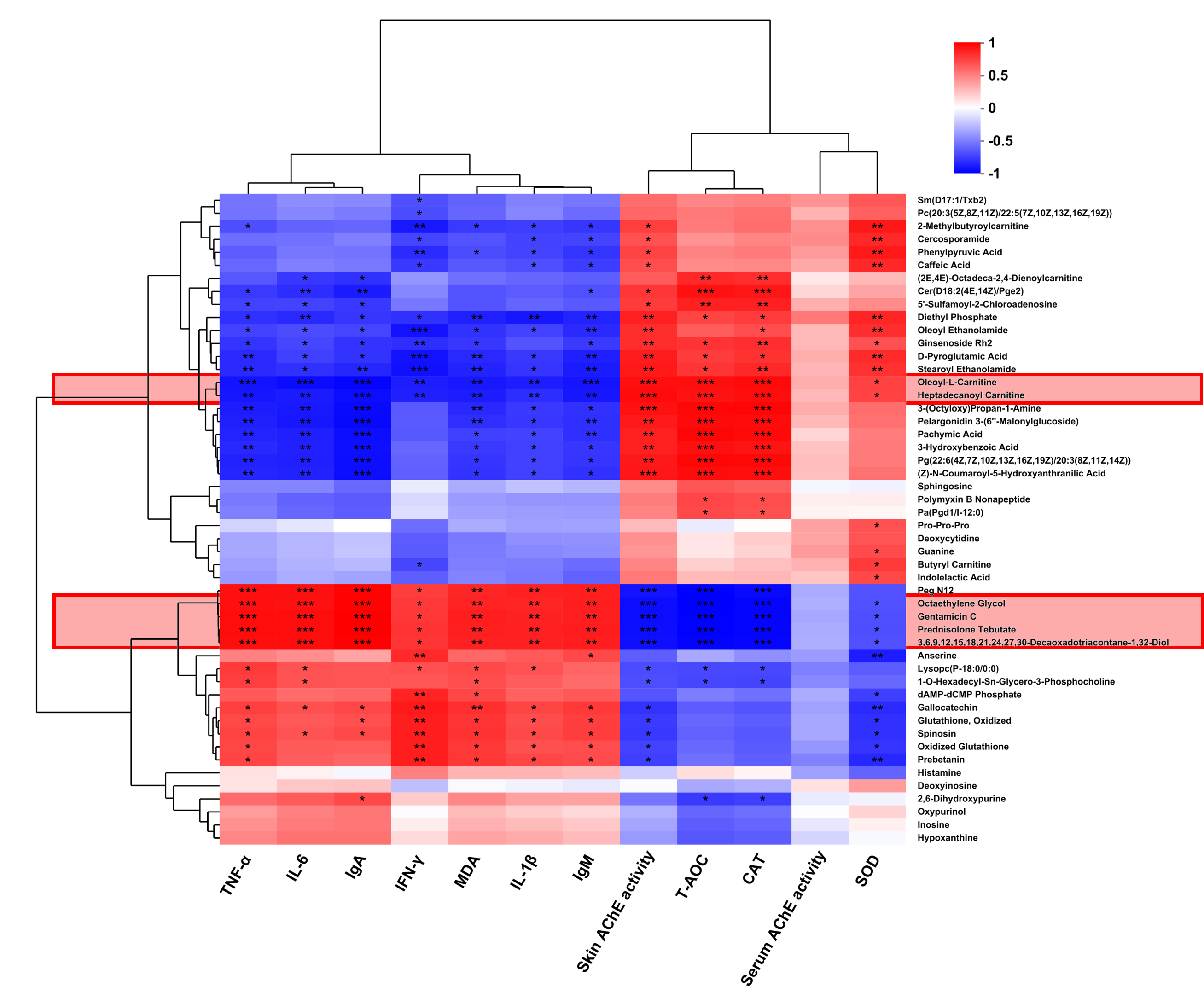


**Fig. S7.** Correlation heatmap between metabolic biomarkers and characteristic biochemical indicators.
